# Investigating the Role of *TNFSF12* in Thyroid Cancer Progression via Single‐Cell RNA Sequencing and Integrated Multiomics Analyses

**DOI:** 10.1155/mi/4753653

**Published:** 2026-04-03

**Authors:** Junjie Yu, Jingjing Li, Shengnan Gao, Lilan Wang, Hong Qiao

**Affiliations:** ^1^ Department of Endocrinology, The Second Affiliated Hospital of Harbin Medical University, Harbin, 150081, Heilongjiang Province, China, hrbmush.edu.cn

**Keywords:** *MERTK*, *MSR1*, scRNA-seq, thyroid cancer, *TNFSF12*

## Abstract

**Background:**

Thyroid carcinoma is characterized by significant heterogeneity and immune evasion, in which myeloid cells play a pivotal role in tumor microenvironment (TME) remodeling. However, the key regulatory genes and their underlying mechanisms are not yet fully elucidated.

**Objective:**

This study aims to identify and validate critical myeloid‐derived genes involved in driving thyroid cancer progression by employing an integrated multiomics approach.

**Methods:**

We performed single‐cell RNA sequencing (scRNA‐seq) on thyroid cancer tissues, integrated with High‐dimensional weighted gene co‐expression network analysis (hdWGCNA) for coexpression module identification, Mendelian randomization (MR) for causal inference, and functional validation assays in thyroid carcinoma cell lines including qPCR, CCK‐8, colony formation, and transwell assays. Microbial correlation analysis and molecular docking were additionally conducted to explore potential interactions.

**Results:**

We identified a tumor‐specific myeloid subpopulation (C5) and a disease‐associated coexpression module harboring three hub genes: *MERTK*, *MSR1*, and *TNFSF12*. MR analysis confirmed *MERTK* and *MSR1* as genetic risk factors for thyroid cancer progression, whereas *TNFSF12* exhibited protective effects. Functional experiments demonstrated that *TNFSF12* enhances proliferative, invasive, and migratory capacities in thyroid cancer cells. Furthermore, *MSR1* and *MERTK* were found to be significantly correlated with specific intratumoral microbiota and associated with BRAF mutation and response to immunotherapy.

**Conclusion:**

Our study reveals a myeloid‐centered regulatory network in thyroid cancer and highlights *TNFSF12* as a context‐dependent oncogene, offering novel insights into targeted therapy and immunotherapeutic strategies.

## 1. Introduction

Thyroid cancer, characterized by the malignant transformation of follicular or parafollicular thyroid cells, arises from a complex interplay between genetic aberrations and environmental factors [1]. The primary pathogenic mechanisms include somatic driver gene mutations (e.g., *BRAF*, *RAS*, *RET/PTC*), epigenetic dysregulation, and exposure to ionizing radiation—particularly during childhood. Epidemiologically, the disease exhibits a marked female predominance (female‐to‐male ratio = 3:1), with peak incidence occurring in early to middle adulthood (ages 30–50 years) [2]. Incidence rates vary significantly across geographic regions and are further elevated in populations with prior exposure to radioactive iodine (e.g., post‐Chernobyl cohorts, individuals receiving therapeutic radiotherapy) [3]. Early detection through ultrasonography combined with fine‐needle aspiration cytology markedly improves prognostic outcomes, highlighting the imperative of timely clinical intervention.

Contemporary therapeutic strategies are tailored according to histopathological subtype (e.g., papillary, follicular, medullary, anaplastic) and tumor stage [4]. Primary treatment typically begins with surgical resection, ranging from lobectomy to total thyroidectomy, often followed by adjuvant radioactive iodine (^131^I) ablation in patients with iodine‐avid differentiated carcinomas [4, 5]. Lifelong thyroid hormone replacement therapy is required to maintain euthyroidism and suppress thyrotropin (TSH)‐driven neoplastic proliferation. Prognostic assessment relies on dynamic risk stratification, incorporating serial postoperative measurements of serum thyroglobulin (Tg), anti‐Tg antibodies, cervical ultrasonography, and, when clinically indicated, diagnostic (^131^I) scintigraphy. Rigorous long‐term surveillance protocols—spanning years to decades—are essential for the early detection of recurrent or metastatic disease, enabling timely therapeutic adjustments to reduce morbidity and mortality [6, 7].

The rapidly advancing field of bioinformatics has profoundly transformed oncogenomics by providing sophisticated computational frameworks for deciphering disease pathogenesis. High‐throughput technologies, particularly single‐cell RNA sequencing (scRNA‐seq), enable the characterization of transcriptional heterogeneity within tumor microenvironments (TMEs) and the identification of rare malignant subclones associated with therapeutic resistance and metastatic spread [8]. Complementarily, gene expression microarrays facilitate genome‐wide profiling of aberrantly expressed transcripts, splice variants, and noncoding RNAs across large patient cohorts [9, 10]. Integrated bioinformatic analysis of multiomics datasets—including differential expression, pathway enrichment, protein–protein interaction network mapping, and survival correlation analyses—systematically prioritizes key oncogenic drivers, tumor suppressors, and druggable targets. These molecular signatures reveal novel pathogenic pathways, refine diagnostic and prognostic biomarkers, and ultimately support the rational development of targeted therapeutics for personalized management of thyroid cancer.

Notably, the *TNFSF12* (also known as *TWEAK*) and its receptor *TNFRSF12A* (*Fn14*) signaling axis has been implicated in inflammation, tissue regeneration, and cancer, exhibiting context‐dependent pro‐ or antitumor effects across different malignancies.

Therefore, this study aimed to: (1) characterize tumor‐associated myeloid (TAM) subpopulations in thyroid cancer using scRNA‐seq; (2) identify causal hub genes within these subpopulations by integrating high‐dimensional weighted gene co‐expression network analysis (hdWGCNA) and Mendelian randomization (MR); and (3) functionally validate the role of a core gene through in vitro assays and explore its multiomics associations with the TME, intratumoral microbiome, and clinical outcomes.

## 2. Materials and Methods

### 2.1. Data Sources

#### 2.1.1. scRNA‐seq

Publicly available single‐cell RNA‐seq data of thyroid cancer patients (Accession: GSE241184) were downloaded from the Gene Expression Omnibus (GEO) database (www.ncbi.nlm.nih.gov/geo). In GSE241184, the analysis was conducted on the tumor site tissues of GSM7718959 and the adjacent tissues of GSM7718960.

#### 2.1.2. Bulk Transcriptomic Data

RNA sequencing data and corresponding clinical information for 572 patients with thyroid carcinoma were obtained from The Cancer Genome Atlas Thyroid Cancer (TCGA‐THCA) collection.

#### 2.1.3. Genetic Data

Summary statistics for expression quantitative trait loci (eQTL) were sourced from the eQTLGen consortium. Genome‐wide association study (GWAS) summary statistics for thyroid cancer were acquired from the GWAS Catalog (Accession: ebi‐a‐GCST90018709).

### 2.2. Single‐Cell Data Processing and Analysis

Single‐cell data analysis was conducted using the Seurat package (v4.x) in R. Low‐quality cells were filtered out based on thresholds for unique molecular identifiers (UMIs), the number of detected genes, and mitochondrial gene percentage (using a median absolute deviation of 3). Potential doublets were identified and removed using DoubletFinder (v2.0.4). Gene expression counts were log‐normalized, and cell cycle effects were regressed out. Technical variations between samples were corrected using the harmony algorithm. Cell types were annotated by referencing canonical marker genes documented in the CellMarker database and published literature.

#### 2.2.1. Coexpression Network and Causal Inference Analysis hdWGCNA

hdWGCNA was performed on the filtered single‐cell data to identify gene modules. A soft‐thresholding power of 8 was selected to construct a scale‐free coexpression network.

#### 2.2.2. MR

Independent cis‐eQTLs (*p*  < 1 × 10^−5^; located within ±1 Mb of the transcriptional start site) for the candidate genes were used as instrumental variables. The validity of this MR analysis relies on three core assumptions for the instrumental variables: (1) relevance (strong association with the exposure, i.e., gene expression), (2) independence (no association with confounders of the exposure–outcome relationship), and (3) exclusion restriction (affecting the outcome only through the exposure and not via alternative pathways). The causal relationship with thyroid cancer risk was primarily estimated using the inverse‐variance weighted (IVW) method. Robustness of the results was assessed through supplementary methods, including MR‐Egger, weighted median, and weighted mode estimators. Sensitivity analyses, such as Cochran’s *Q* test and leave‐one‐out analysis, were performed to evaluate heterogeneity and potential pleiotropy.

### 2.3. Functional Enrichment and Multiomics Profiling

Pathway enrichment analysis was performed using gene set enrichment analysis (GSEA) and gene set variation analysis (GSVA) with gene sets from the MSigDB database. Significantly enriched pathways were defined as those with a false discovery rate (FDR)‐adjusted *p*‐value <0.05. Tumor mutational burden (TMB) was quantified as the number of nonsynonymous mutations per megabase, and neoantigens were predicted from somatic mutations using NetMHCpan 4.0. Intratumoral microbiome composition data were analyzed using a standardized bioinformatics pipeline incorporating taxonomic classification, decontamination, and abundance quantification. Cell–cell communication networks were inferred from single‐cell data using CellCall, and developmental trajectories were constructed using monocle.

### 2.4. Molecular Docking

Protein structures for target genes were retrieved from the RCSB Protein Data Bank or predicted using AlphaFold. Small molecule compounds were identified through the Comparative Toxicogenomics Database, and their 3D structures were obtained from PubChem. Molecular docking simulations were performed using AutoDock, with 50 independent runs per ligand–protein pair to sample conformational space. The pose with the most favorable binding energy was selected, and molecular interactions were visualized using PyMOL.

### 2.5. Clinical Prediction Modeling

To generate an individualized prognostic tool, we developed a nomogram that integrates selected gene expression signatures with key clinical variables. The nomogram was constructed based on the coefficients of a multivariable Cox proportional hazards regression model, translating the contribution of each predictor into a point‐based scoring system. The total points project to a probability of clinical outcome, aiding in risk stratification and clinical decision‐making.

### 2.6. Cell Culture and Transfection

The experiments were conducted using three papillary thyroid carcinoma cell lines (*TPC-1*, *BCPAP*, and *IHH4*) and one normal thyroid epithelial line (Nthy‐ori 3‐1). Nthy‐ori 3‐1 cells were purchased from Shanghai Jinyuan Biotechnology Co., Ltd. (human, normal thyroid epithelium; RRID: CVCL_3857, Cat.no. 240625B‐1); BCPAP cells were purchased from Shanghai Jinyuan Biotechnology Co., Ltd. (human, papillary thyroid carcinoma; RRID: CVCL_0153, Cat.no. 250926D); TPC‐1 cells were purchased from Shanghai Jinyuan Biotechnology Co., Ltd. (human, papillary thyroid carcinoma; RRID: CVCL_6293, Cat.no. 240808S) and IHH4 cells were purchased from Wuhan Procell Life Technology Co., Ltd. (human, papillary thyroid carcinoma; RRID: CVCL_3494, Cat.no. CL‐0803). All cell lines were cultured in RPMI‐1640 medium containing 10% heat‐inactivated fetal bovine serum, under a humidified atmosphere at 37°C with 5% CO_2_. Cell line authentication was performed using short tandem repeat (STR) profiling (SiFaSTR 23 plex Kit, ABI Prism 3130 XL). STR profiling confirmed the identity of all cell lines: Nthy‐ori 3‐1 and TPC‐1 matched their respective ExPASy reference profiles (100% at 13 core STR loci). BCPAP matched the B‐CPAP reference profile, and IHH4 matched its specific reference profile (both 100% match). All cell lines were confirmed to be free of mycoplasma contamination using PCR‐based testing prior to experimental use. TNFSF12 overexpression employed pcDNA3.1 vector containing the full‐length coding sequence. For knockdown, we used synthetic siRNA targeting *TNFSF12* (sequences in Supporting Information: Table S1). Thyroid cancer cells were transfected at 50 nM oligonucleotide concentration using Lipofectamine 2000 according to the manufacturer’s protocol, with 48–72 h allowed for maximal knockdown or overexpression before functional assays.

### 2.7. Quantitative PCR

Total RNA was isolated using TRIzol reagent according to the standard phenol‐chloroform method. RNA integrity and concentration were evaluated by NanoDrop spectrophotometry, and samples with A260/A280 ratios ranging from 1.8 to 2.0 were considered acceptable. One microgram of total RNA was reverse transcribed using the PrimeScript RT reagent kit with a mix of oligo‐dT and random hexamer primers.

Real‐time PCR was performed using SYBR Green chemistry and gene‐specific primers (sequences provided in Supporting Information: Table S2). Gene expression levels were normalized to GAPDH as an endogenous control, with relative quantification calculated using the 2^-ᐃᐃᶜᵗ^ method.

### 2.8. CCK‐8 Viability Assay

Cells were seeded at a density of 5000 cells per well in 96‐well plates. At predetermined time points (12, 24, 48, and 72 h), CCK‐8 reagent was added, and plates were incubated for 2 h. Absorbance was measured at 450 nm using a microplate reader, with values proportional to viable cell number.

### 2.9. Colony Formation

Twenty‐four hours after transfection, cells were reseeded at low density (800 cells per 3.5 cm dish) and cultured for ~14 days, with the culture medium replaced every 48 h. Visible colonies were fixed with 4% paraformaldehyde, stained with 0.1% crystal violet, and manually counted. Colonies consisting of ≥50 cells were defined as positive, and three random selected fields were quantified per dish.

### 2.10. Transwell Invasion

Transfected cells (4 × 10^5^) were seeded into the upper chamber of 8 μm pore transwell inserts in serum‐free medium. The lower chamber was filled with medium containing 20% FBS as chemoattractant. Following 24‐h incubation, nonmigrated cells on the upper surface were removed using cotton swabs. Cells that had migrated through the membrane were fixed, stained with crystal violet, and quantified by counting in five randomly selected microscopic fields.

### 2.11. Wound Healing Assay

Confluent cell monolayers in six‐well plates were scratched using a 200 μL pipette tip to create a uniform wound. Detached cells were removed by washing with PBS, and the initial wound width was recorded by photography. Migration distance was quantified by comparing images acquired at 24 h postscratch with baseline images.

### 2.12. Statistical Analysis

All computational and statistical analyses were performed in R (v4.3.2). Data from in vitro experiments are presented as the mean ± standard deviation (SD) from at least three independent biological replicates. Group differences were analyzed using a two‐tailed Student’s *t*‐test (for two groups) or one‐way ANOVA (for multiple groups). A FDR‐adjusted *p*‐value (Benjamini–Hochberg method) of less than 0.05 was considered statistically significant. MR was performed using the TwoSampleMR package (v0.5.7). hdWGCNA was conducted using the hdWGCNA package (v1.0.0).

## 3. Results

### 3.1. A TAM Subpopulation Shapes the Immunosuppressive Microenvironment

scRNA‐seq of two thyroid carcinoma samples, after rigorous quality control and doublet removal, yielded 13,880 high‐quality cells for analysis (Figure 1A–C). Unsupervised clustering of the integrated data identified 12 major cell types within the TME (Figure 2A–E), which were annotated into 10 major cell types based on the expression of canonical marker genes (Figure 2F). Myeloid cells were significantly enriched in tumor tissues (Figure 2G). Reclustering of the myeloid compartment revealed eight distinct subpopulations (Figure 2H–L). Among these, the C5 subpopulation was specifically enriched in tumors and was thus defined as a TAM subset (Figure 2M). This TAM subset comprised three synergistic cellular states: dendritic cells (high *HLA-DRA*, *HLA-DPB1*), immunosuppressive macrophages (high *CD163*, *LYZ*), and inflammatory monocytes (high *S100A8/A9*), as evidenced by the marker gene expression (Figure 2N–O). These components collectively form a protumorigenic niche.

Figure 1Quality control metrics for scRNA‐seq data processing. (A) Distribution of QC metrics: violin plots show nCount_RNA (total UMIs), nFeature_RNA (genes detected), and percent.mt (mitochondrial reads) distributions for samples GSE241184. (B) Correlation validation: scatterplots confirm strong positive correlation between genes and UMIs (*r* = 0.97), and minimal correlation between mitochondrial content and UMIs (*r* = –0.08). (C) Highly variable genes: top 10 genes with greatest standardized variance (e.g., HBA2, CCL21) identified across cells.

**Figure:**
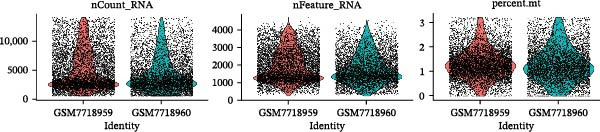
(A)

**Figure:**
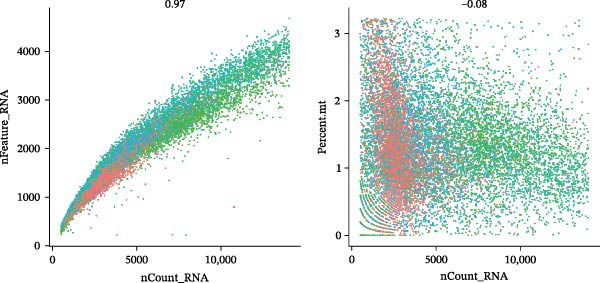
(B)

**Figure:**
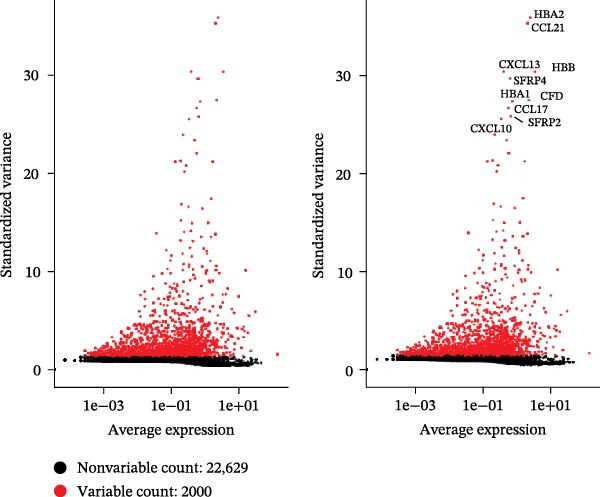
(C)

Figure 2Single‐cell RNA sequencing analysis of thyroid cancer cellular heterogeneity. (A) PC selection: elbow plot determining optimal principal components (PCs = 20). (B, C) Batch effect correction: PCA plots showing batch effects before (B) and after (C) harmony integration. (D, E) Clustering annotation: UMAP plots showing 12 clusters (D) annotated into 10 cell types (E). (F) Marker validation: bubble plot displaying cell‐type‐specific marker genes. (G) Myeloid enrichment: stacked bar plot confirming myeloid dominance in tumors. (H–L) Myeloid subclustering: secondary UMAPs/heatmaps revealing eight myeloid subpopulations. (M) Tumor‐specific signature: bar plot showing C5 subpopulation enrichment in tumors. (N) C5 subclassification: UMAP subdividing C5 into dendritic cells, macrophages, and monocytes. (O) Subtype markers: dot plot highlighting HLA‐DRA/RGS1 (dendritic), CD163/LYZ (macrophage), and S100A8/S100A9 (monocyte).

**Figure:**
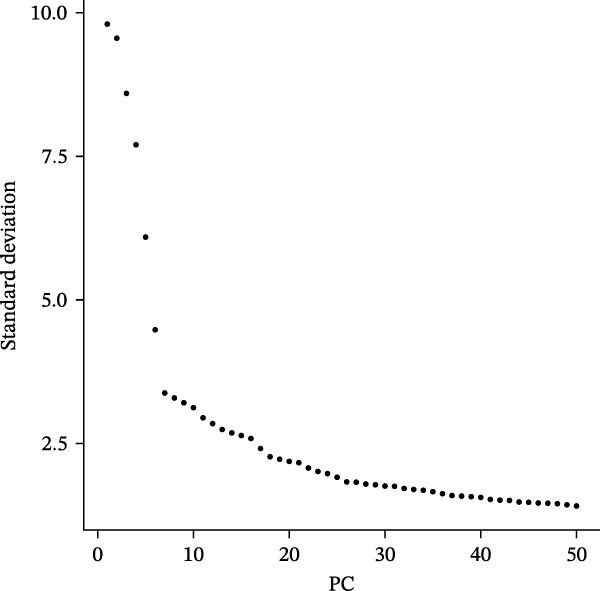
(A)

**Figure:**
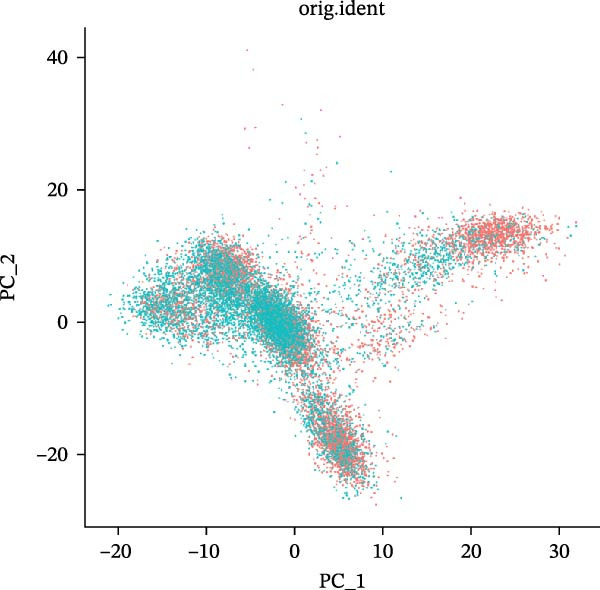
(B)

**Figure:**
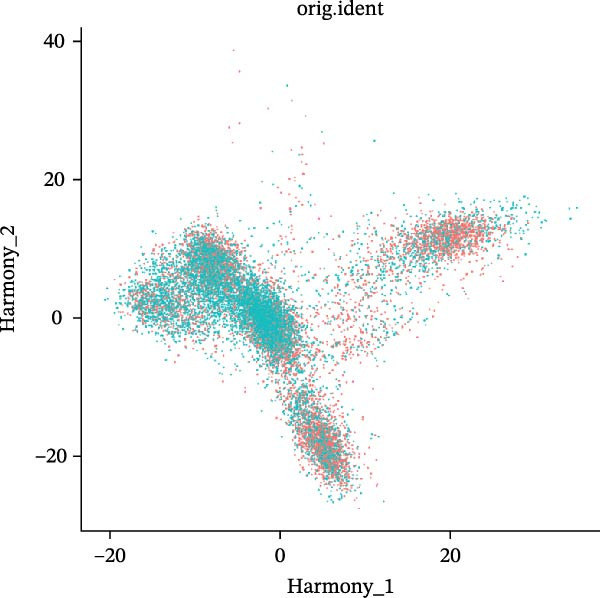
(C)

**Figure:**
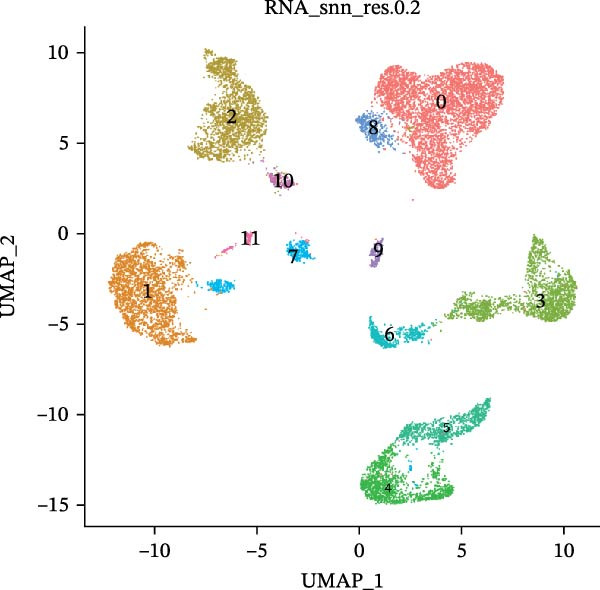
(D)

**Figure:**
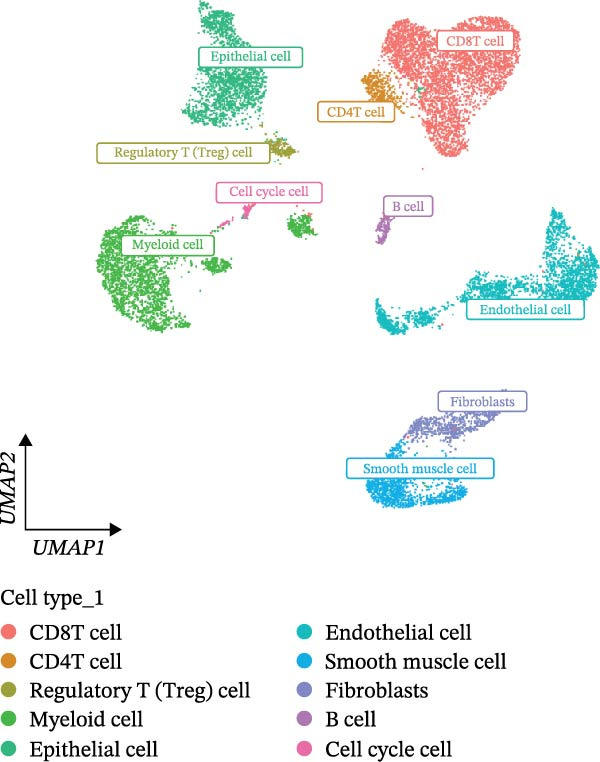
(E)

**Figure:**
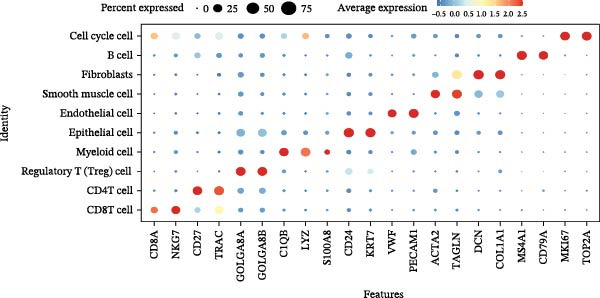
(F)

**Figure:**
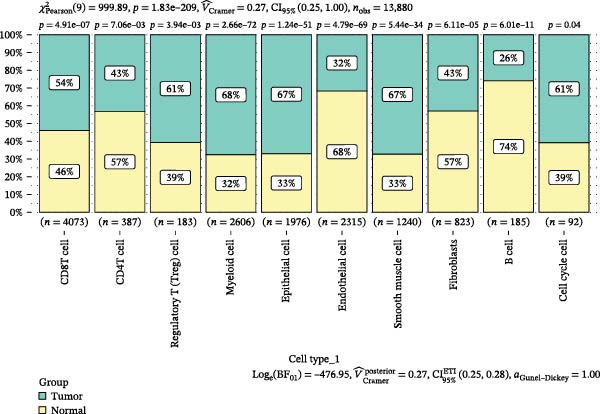
(G)

**Figure:**
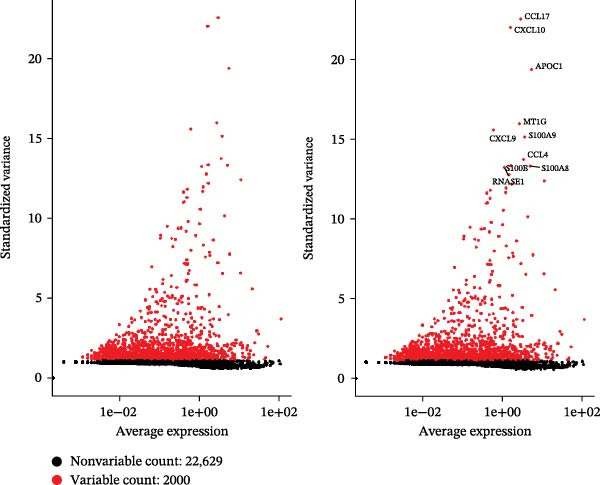
(H)

**Figure:**
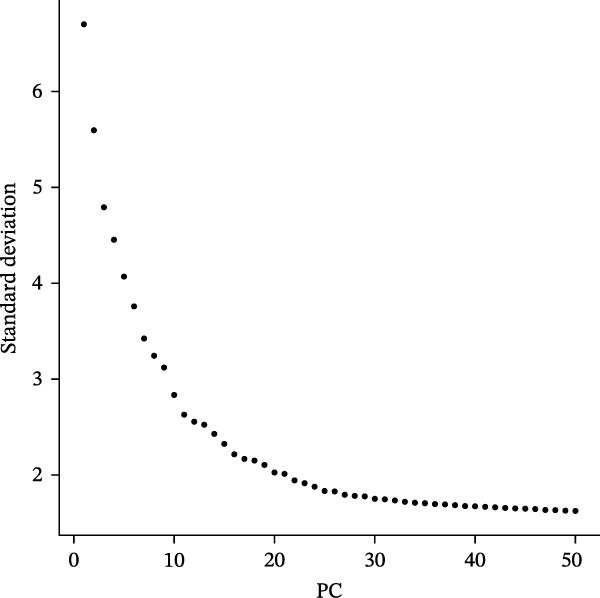
(I)

**Figure:**
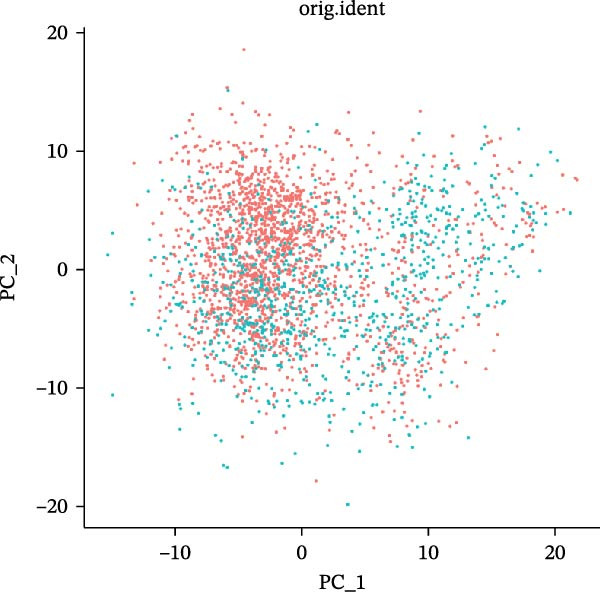
(J)

**Figure:**
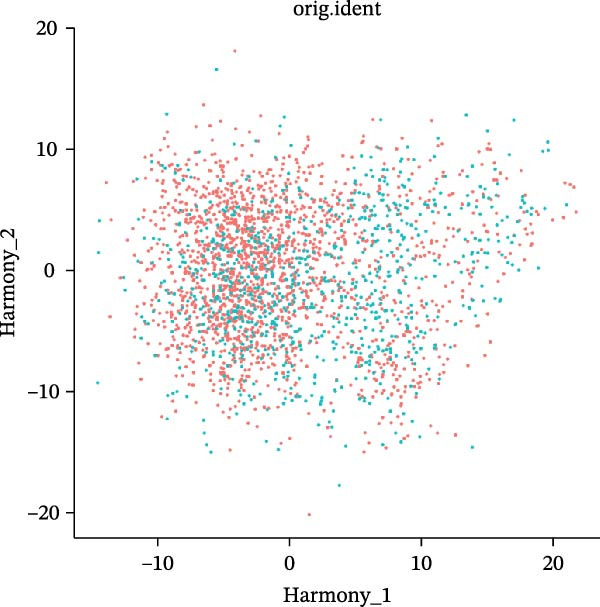
(K)

**Figure:**
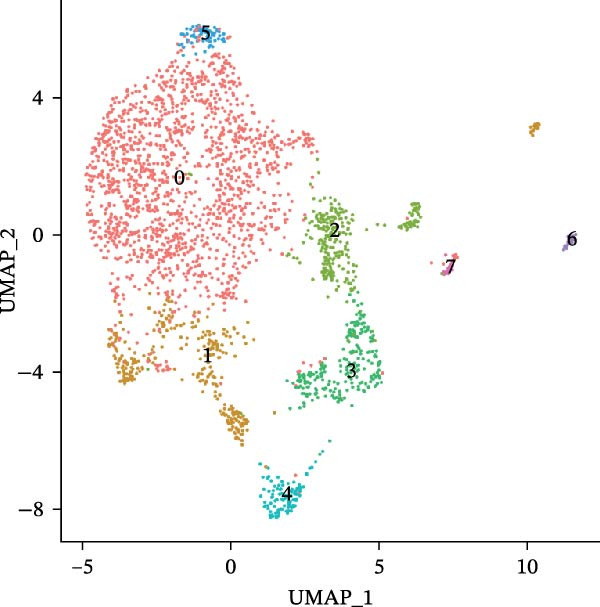
(L)

**Figure:**
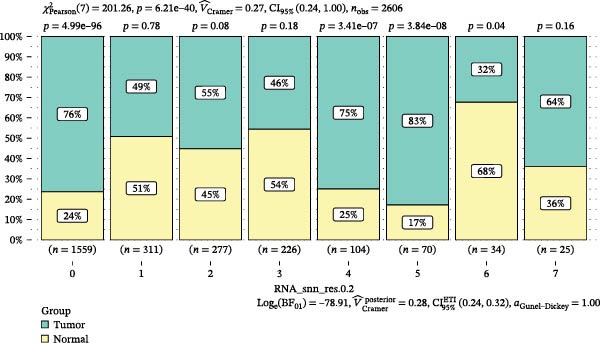
(M)

**Figure:**
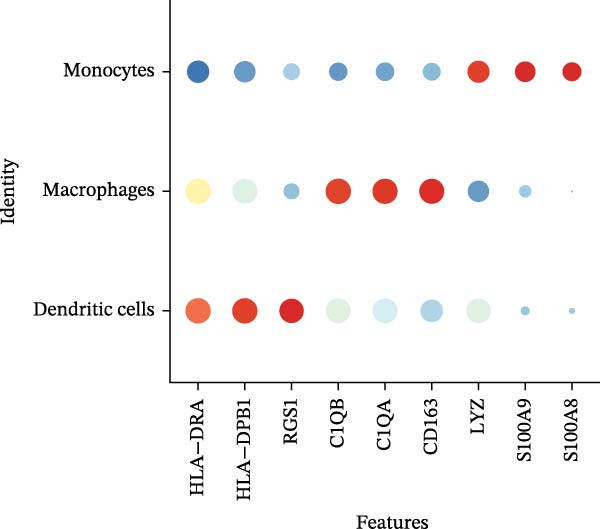
(N)

**Figure:**
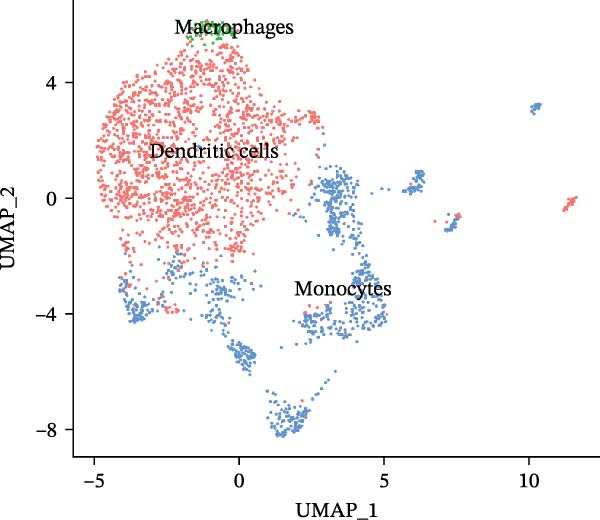
(O)

### 3.2. hdWGCNA and MR Identify Causal Hub Genes

To delineate gene coexpression networks within myeloid subtypes, we performed hdWGCNA. The optimal soft thresholding power (*β* = 8) was determined to ensure a scale‐free topology (Figure 3A). This analysis identified 12 coexpression modules, with their hierarchical relationships depicted in a dendrogram (Figure 3B). Module eigengene (ME) analysis revealed that the yellow module exhibited significantly elevated MEs in TAM subtypes (Figure 3C–E). Consequently, all genes within the yellow module were designated as high‐confidence candidate biomarkers (Supporting Information: Table S3), providing a molecular repository for further exploration.

Figure 3hdWGCNA analysis of gene coexpression networks in myeloid subtypes. (A) Soft‐threshold selection: power *β* = 8 selected to achieve scale‐free topology (*R* ^2^ = 0.85). (B) Module identification: cluster dendrogram of 12 coexpression modules (color‐labeled). (C–E) Tumor‐associated signature: heatmap (C) and eigengene plots (D, E) showing specific upregulation of the yellow module in tumor myeloid subclusters.

**Figure:**
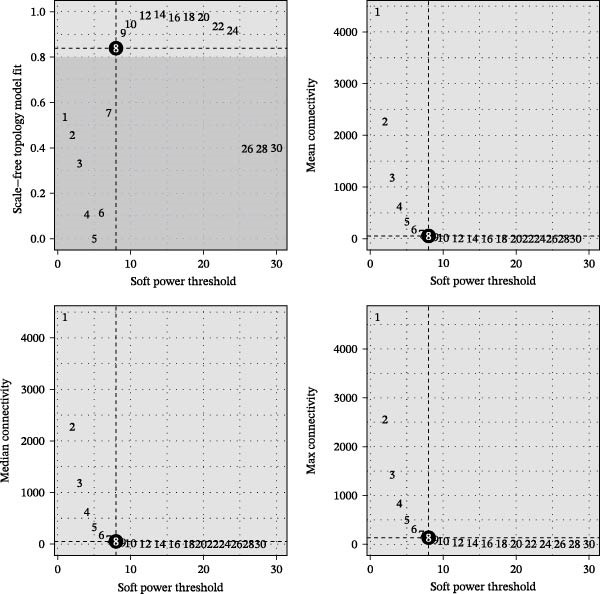
(A)

**Figure:**
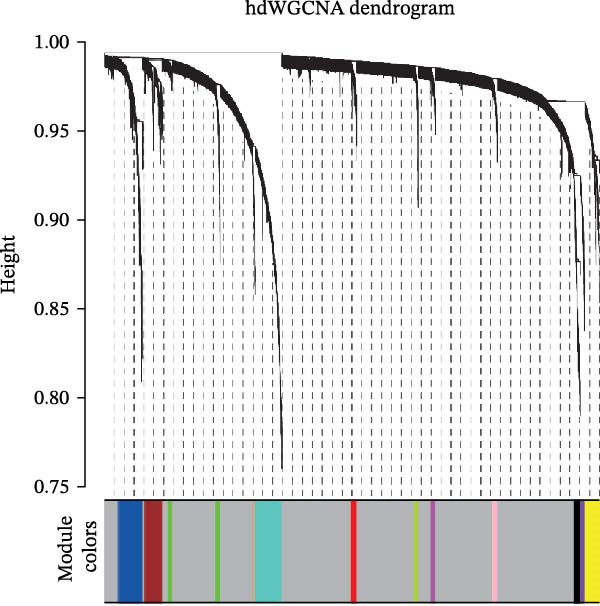
(B)

**Figure:**
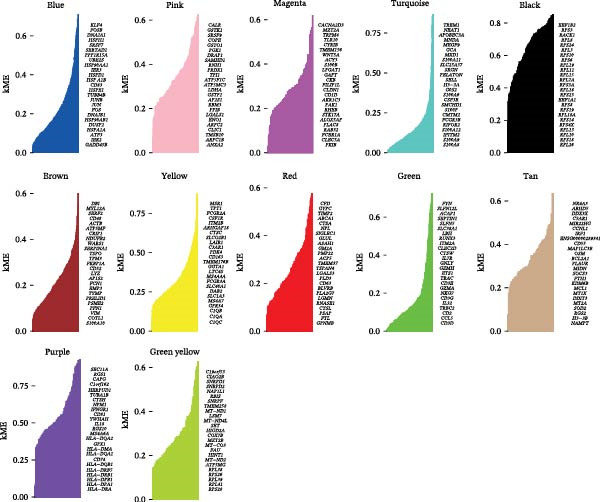
(C)

**Figure:**
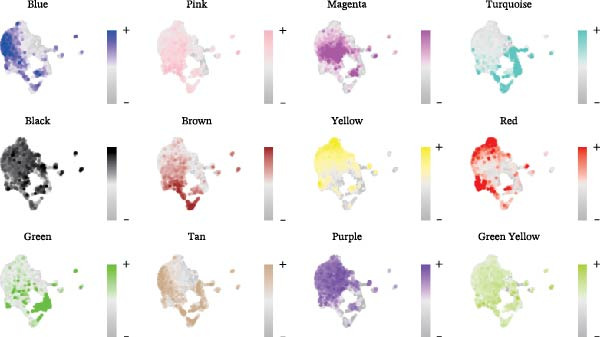
(D)

**Figure:**
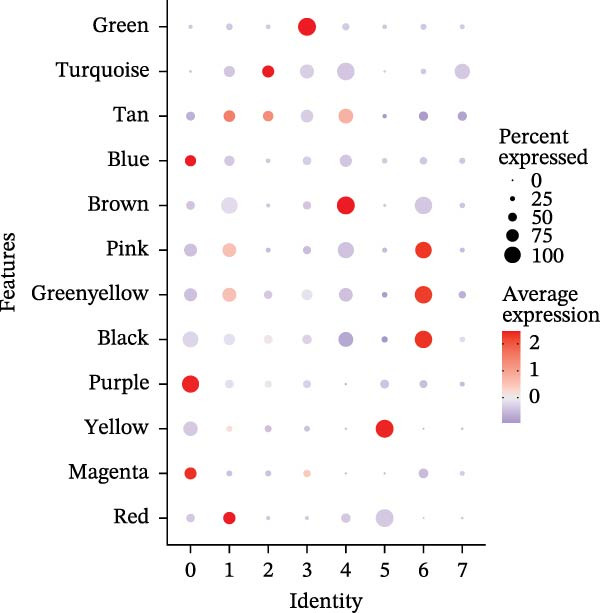
(E)

We then employed two‐sample MR to validate causal relationships for these candidate genes with thyroid cancer risk (Figure 4). The analysis established *MERTK* (OR = 1.47, *p* = 0.049) and *MSR1* (OR = 1.63, *p* = 0.019) as risk factors, and *TNFSF12* (OR = 0.57, *p* = 0.005) as a protective factor, as shown in scatterplots (Figure 4A–C) and forest plots (Figure 4D–F). Sensitivity analyses confirmed the robustness of these causal inferences, with heterogeneity statistics yielding nonsignificant *p*‐values (all >0.05; Supporting Information Table S4), indicating consistent effect estimates across instrumental variable sets.

Figure 4Mendelian randomization validates causal effects of key genes on thyroid cancer risk. (A) *MERTK* causality: scatterplot showing positive IVW slope associating SNP effects with increased risk. (B) *MSR1* causality: scatterplot with steep IVW slope supporting elevated risk. (C) *TNFSF12* causality: scatterplot demonstrating significant protective IVW slope. (D) *MERTK* effect consolidation: forest plot validating aggregate risk without null overlap. (E) *MSR1* effect consolidation: forest plot confirming all SNPs contribute to risk. (F) *TNFSF12* effect consolidation: forest plot maintaining robust protective effect despite individual SNP variations.

**Figure:**
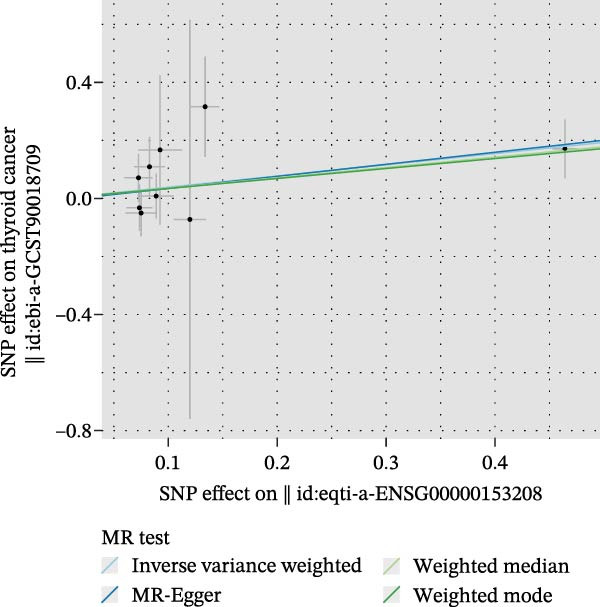
(A)

**Figure:**
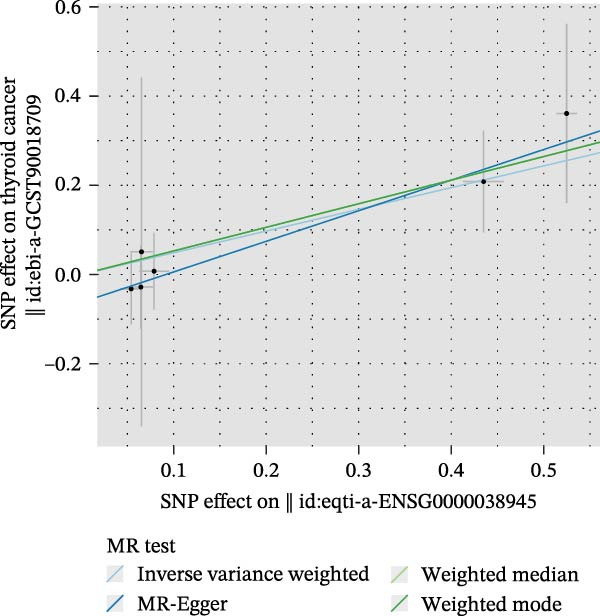
(B)

**Figure:**
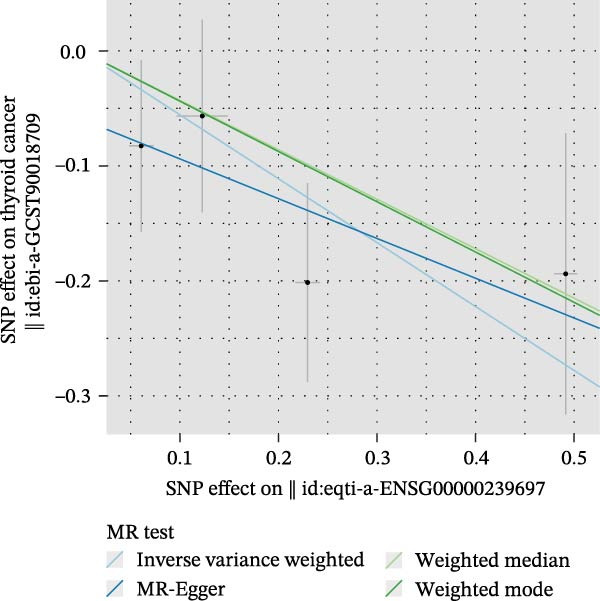
(C)

**Figure:**
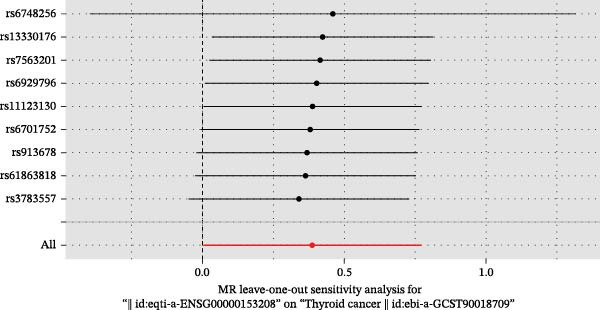
(D)

**Figure:**
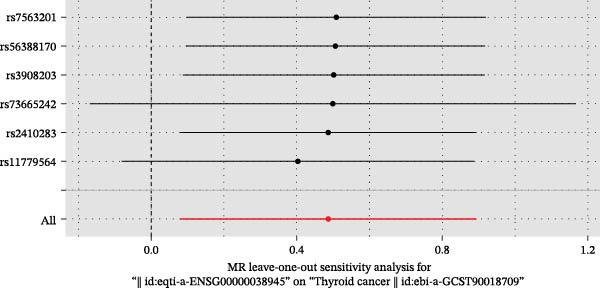
(E)

**Figure:**
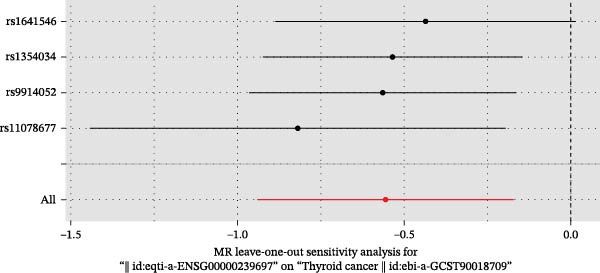
(F)

### 3.3. Key Genes Govern Distinct Oncogenic Pathways and Clinical Features

Functional enrichment analysis revealed that *MERTK*, *MSR1*, and *TNFSF12* regulate distinct oncogenic pathways. Specifically, *MERTK* was linked to chromatin remodeling (e.g., polycomb repressive complex) and cell cycle regulation (e.g., mitotic spindle), *MSR1* to inflammatory and immune responses (e.g., NF‐kappa B signaling, interferon‐gamma response), and *TNFSF12* to metabolic processes (e.g., arachidonic acid metabolism) (Figure 5A–F).

Figure 5Functional enrichment profiles of thyroid cancer hub genes via GSEA and GSVA. (A) *MERTK* GSEA: enriched pathways: polycomb repressive complex, adherens junction, axon guidance. (B) *MSR1* GSEA: enriched pathways: chemokine signaling, NF‐κB signaling, toll‐like receptor signaling. (C) *TNFSF12* GSEA: enriched pathways: chemokine signaling, cytosolic DNA‐sensing, arachidonic acid metabolism. (D) *MERTK* GSVA: bar plot showing WNT_BETA_CATENIN_SIGNALING and MITOTIC_SPINDLE upregulation in Hep group. (E) *MSR1* GSVA: bar plot indicating INTERFERON_GAMMA_RESPONSE and INFLAMMATORY_RESPONSE enrichment in Hep. (F) *TNFSF12* GSVA: bar plot highlighting MYOGENESIS and COAGULATION pathway alterations.

**Figure:**
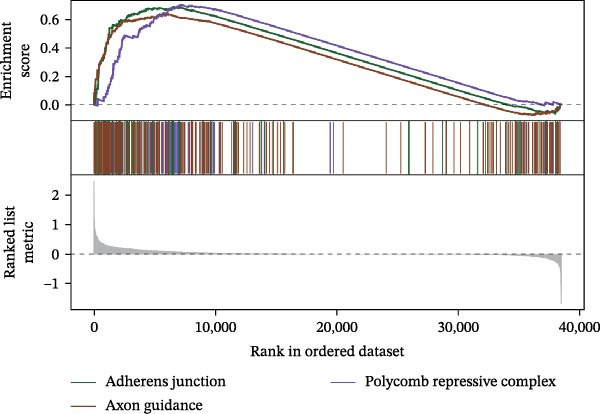
(A)

**Figure:**
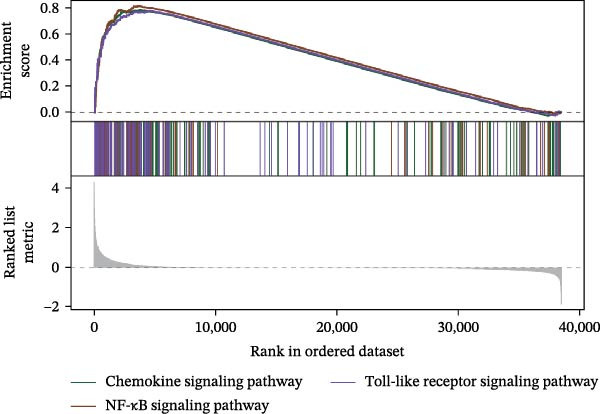
(B)

**Figure:**
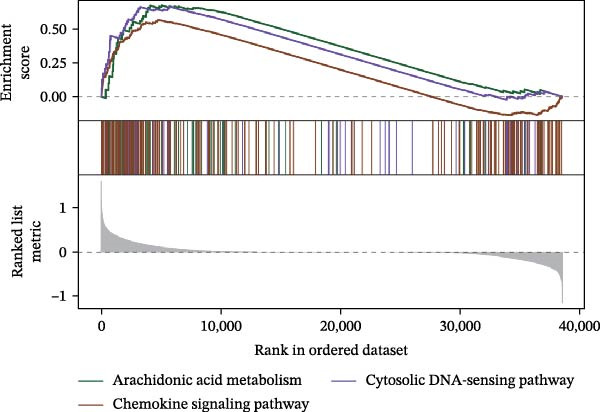
(C)

**Figure:**
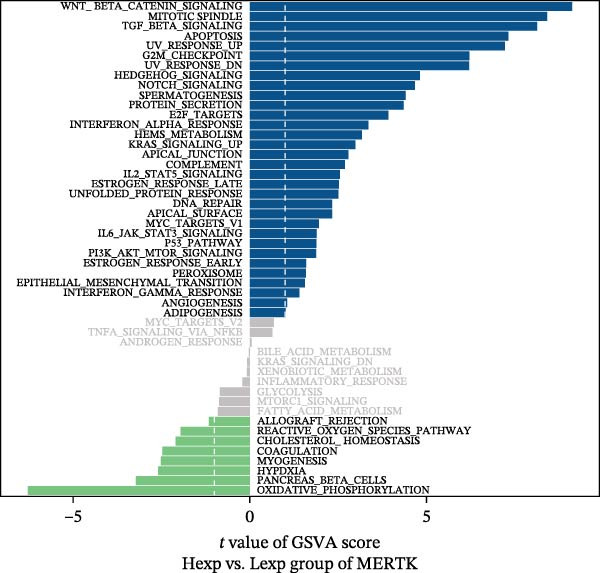
(D)

**Figure:**
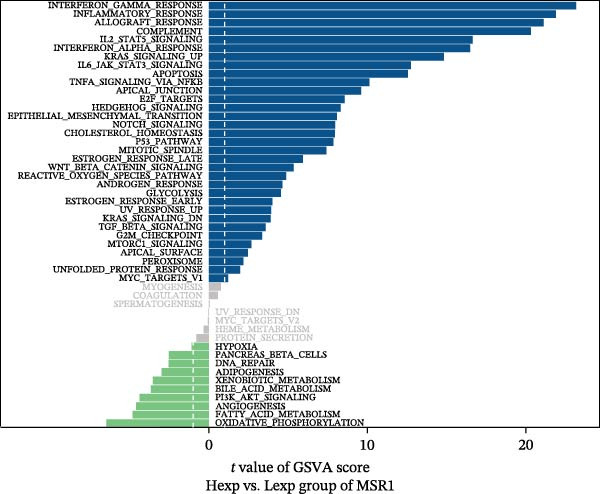
(E)

**Figure:**
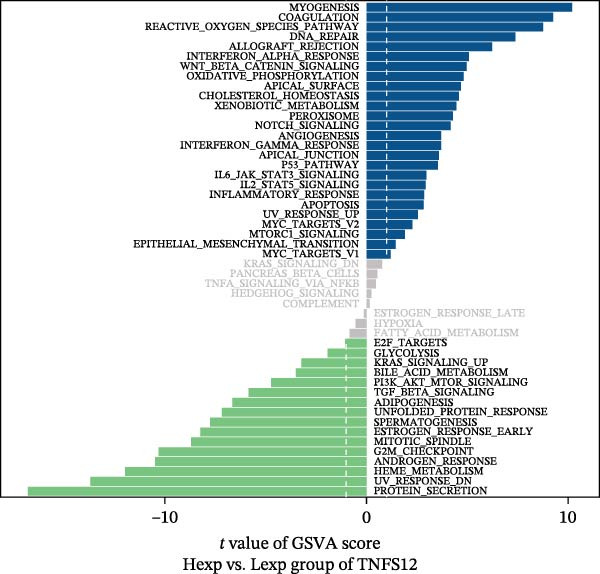
(F)

Clinically, low *TNFSF12* expression correlated with an enhanced immunotherapy response, whereas *MERTK* and *MSR1* showed no significant effect (Figure 6A–C). The mutation landscape revealed higher *BRAF* mutation frequencies in *MERTK*‐high and *MSR1*‐high groups, contrasting with the *TNFSF12*‐low group (Figure 6D–F). Furthermore, *MERTK* expression was associated with increased neoantigen burden and TMB, while *MSR1* correlated only with neoantigen burden; *TNFSF12* showed no significant associations with these biomarkers (Figure 6G–O). We integrated these three genes into a nomogram that effectively predicted patient survival, demonstrating excellent calibration and significant clinical utility in decision curve analysis (Figure 6P–R).

Figure 6Immunotherapeutic and prognostic implications of hub genes in thyroid carcinoma. (A–C) Immunotherapy sensitivity: stacked bar charts showing the proportion of immunotherapy‐sensitive (true) vs. immunotherapy‐insensitive (false) samples across *MERTK*, *MSR1*, and *TNFSF12* expression cohorts (stratified by high/low expression). Among these cohorts, the *TNFSF12*‐low expression group is associated with a higher proportion of immunotherapy‐sensitive samples. (D–F) Mutation landscapes: top 30 gene mutation heatmaps demonstrating elevated BRAF rates in *MERTK*/*MSR1*‐high vs. *TNFSF12*‐low groups. (G‐I) *MERTK* biomarker correlations: violin plots confirming positive associations with neoantigen burden (G) and TMB (I). (J–L) *MSR1* biomarker correlations: significant link to neoantigen burden (J); no MSI/TMB significance. (M–O) *TNFSF12* biomarker correlations: no significant associations with MSI, neoantigens, or TMB. (P) Predictive nomogram: clinical model integrating gene expressions (horizontal length = factor weight) for survival prognosis. (Q) Calibration curves: observed vs. predicted 3‐/5‐year OS probabilities. (R) Clinical utility: decision curve analysis showing net benefit advantage (yellow curve) over “treat all/none” across thresholds.

**Figure:**
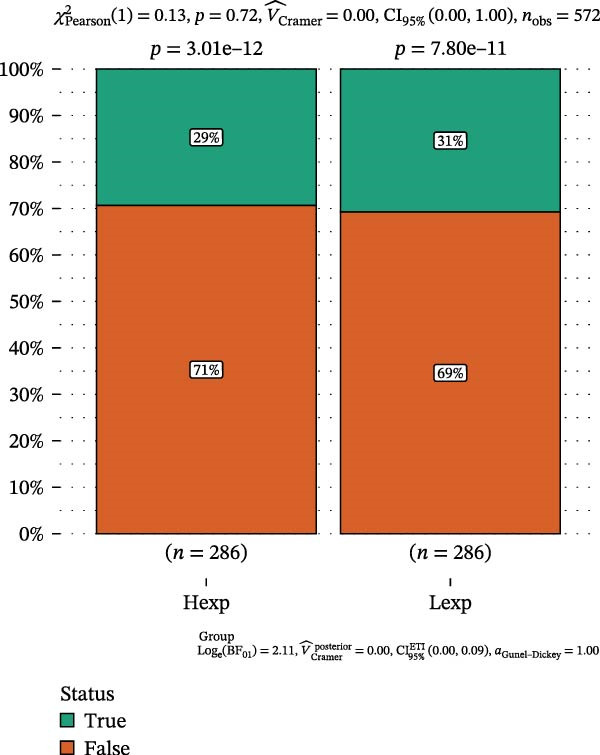
(A)

**Figure:**
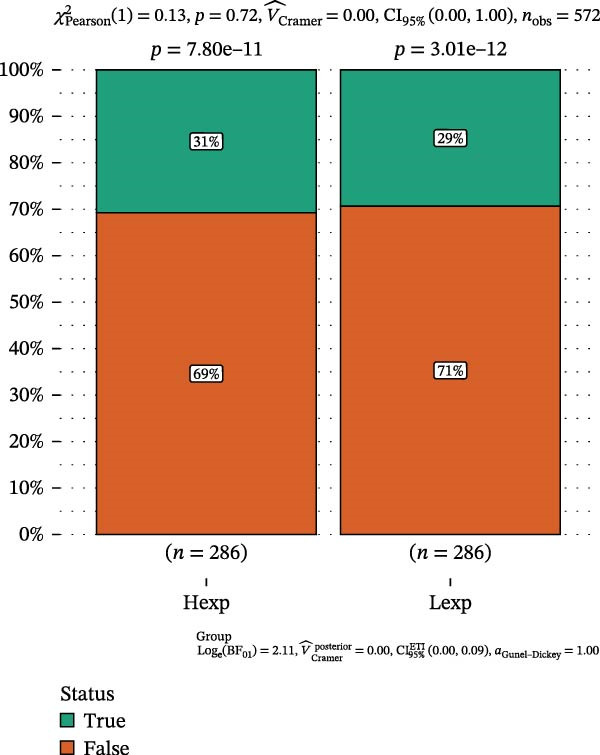
(B)

**Figure:**
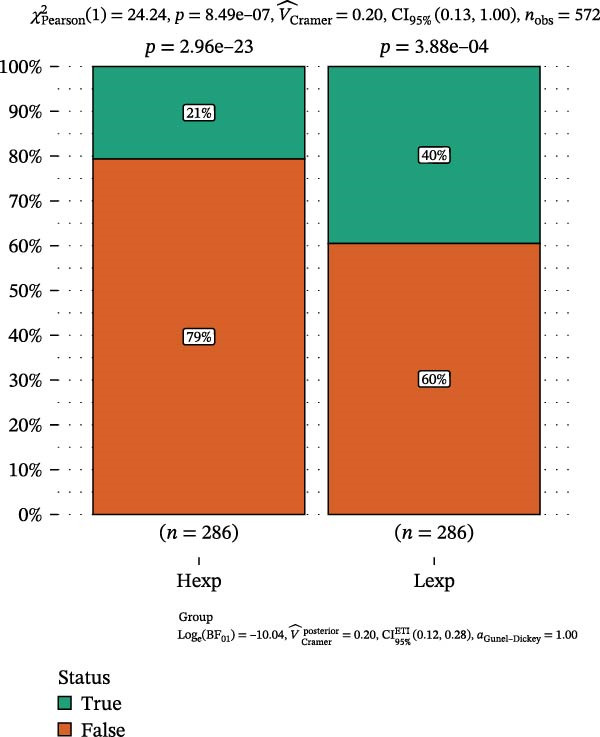
(C)

**Figure:**
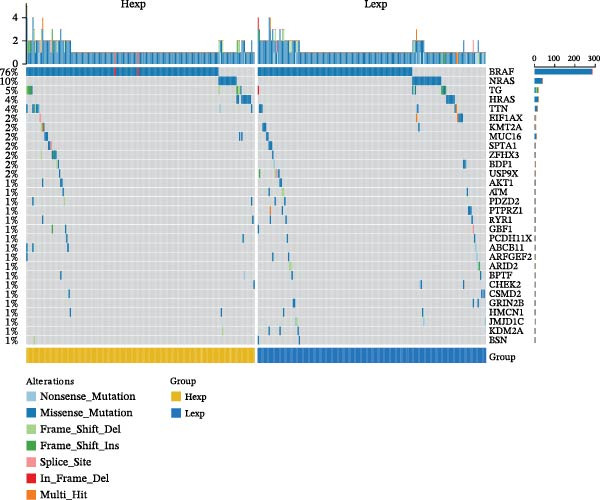
(D)

**Figure:**
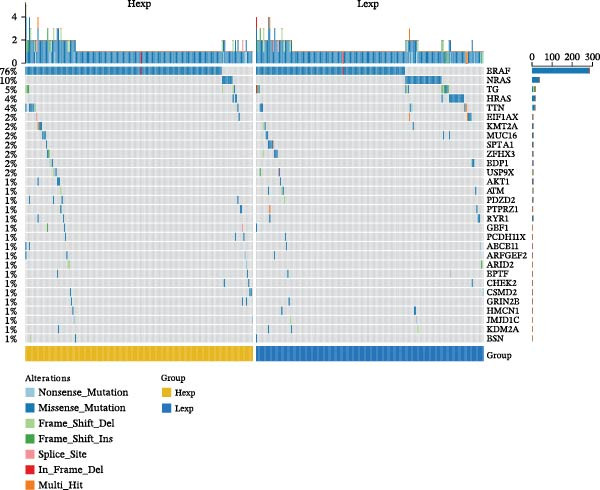
(E)

**Figure:**
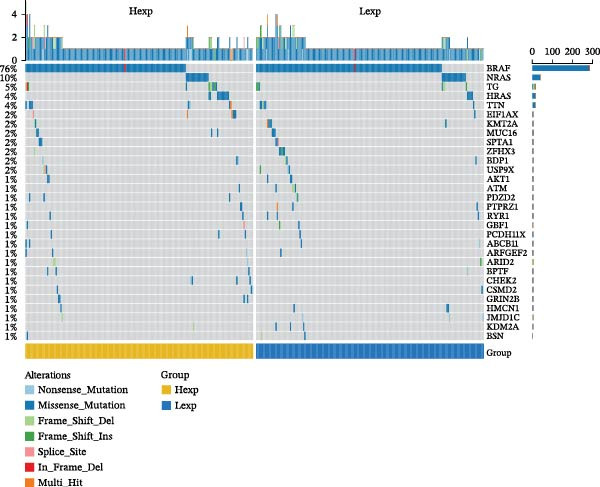
(F)

**Figure:**
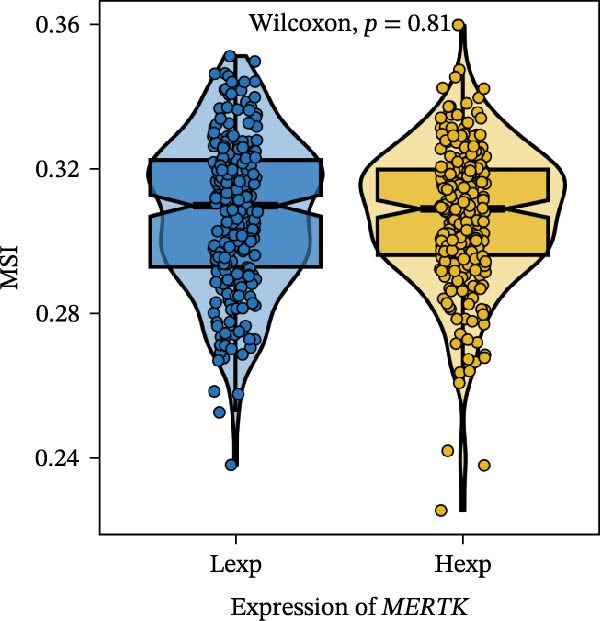
(G)

**Figure:**
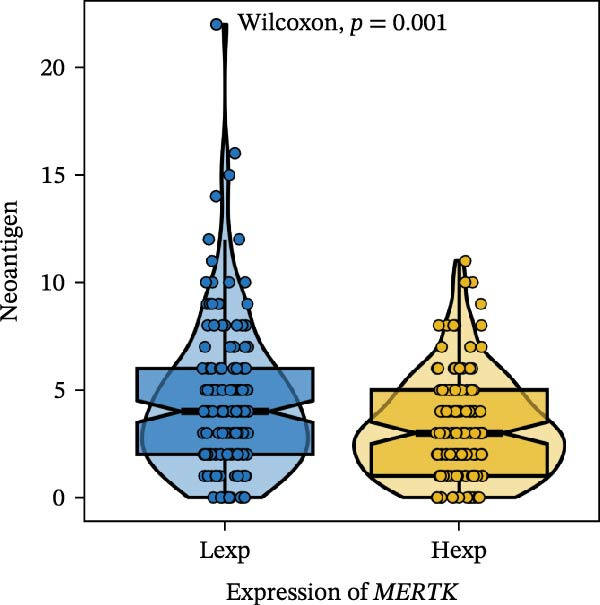
(H)

**Figure:**
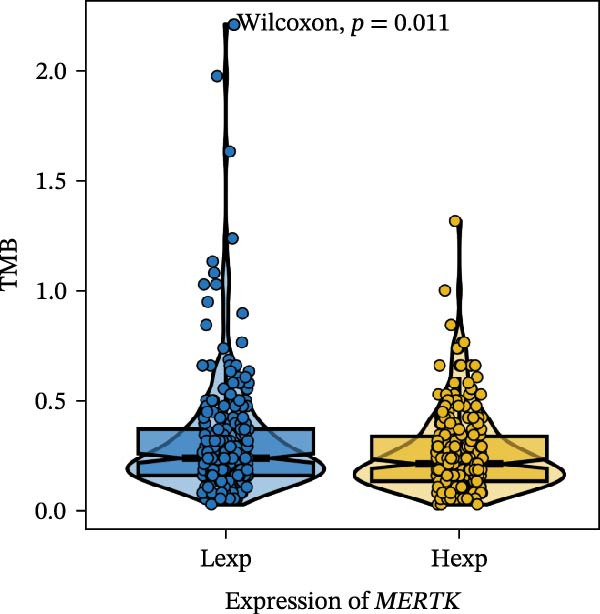
(I)

**Figure:**
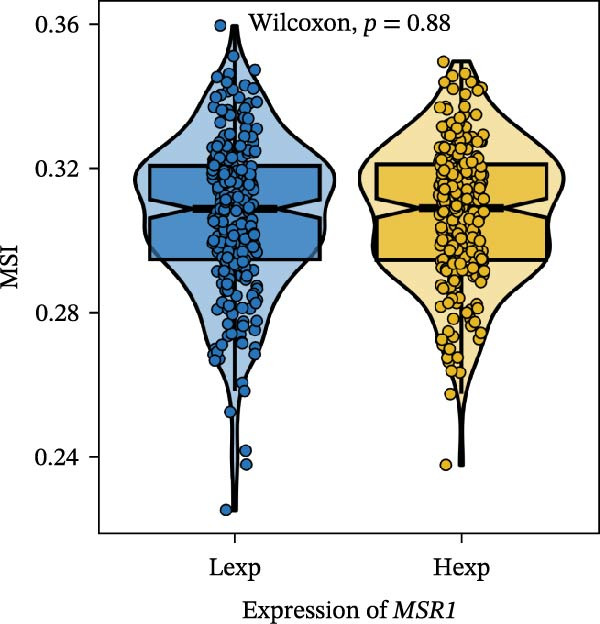
(J)

**Figure:**
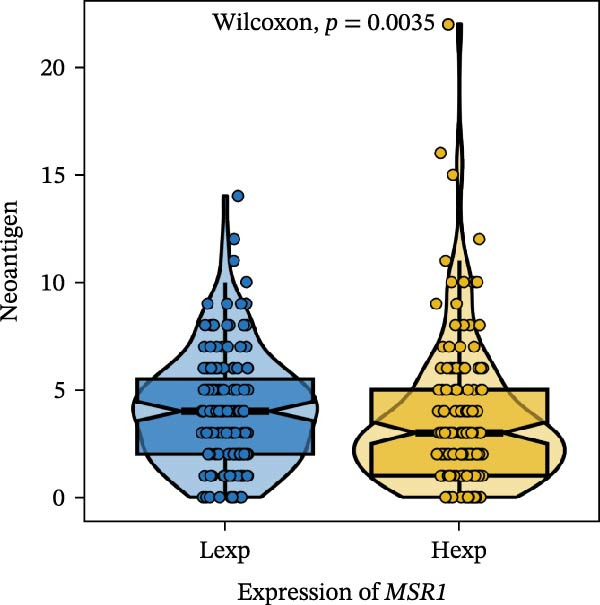
(K)

**Figure:**
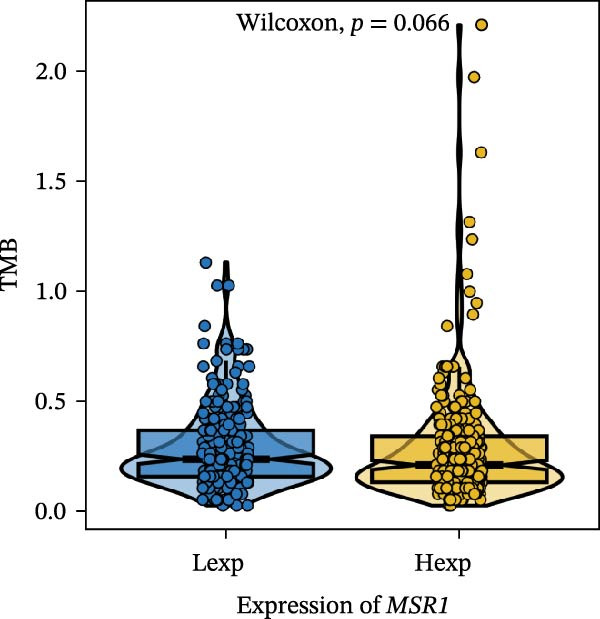
(L)

**Figure:**
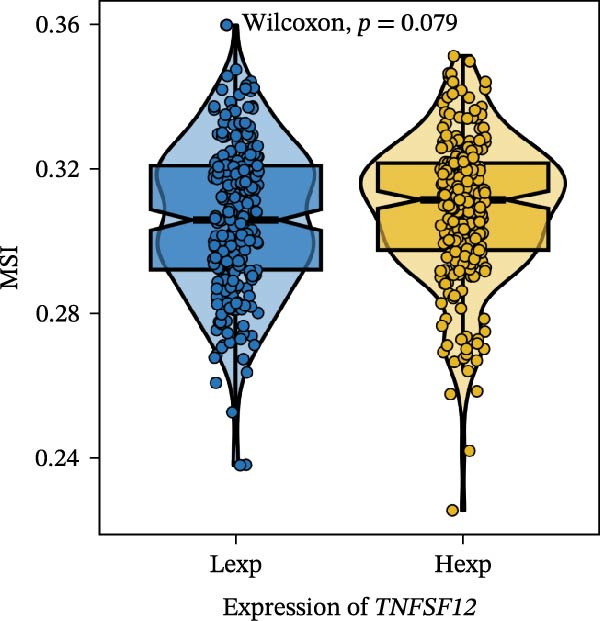
(M)

**Figure:**
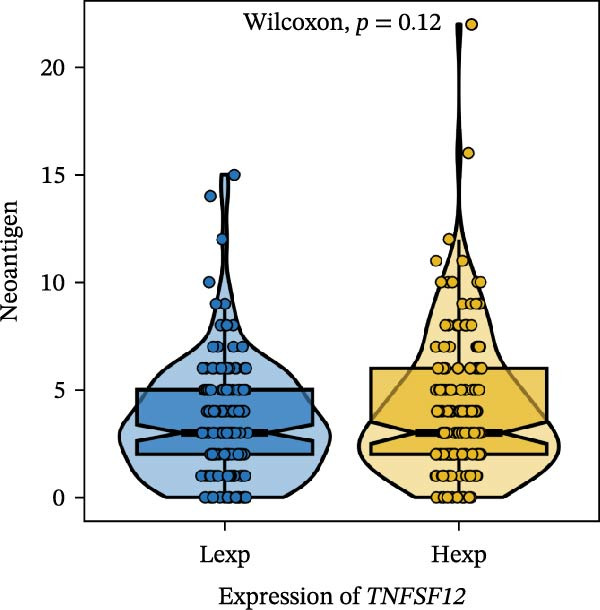
(N)

**Figure:**
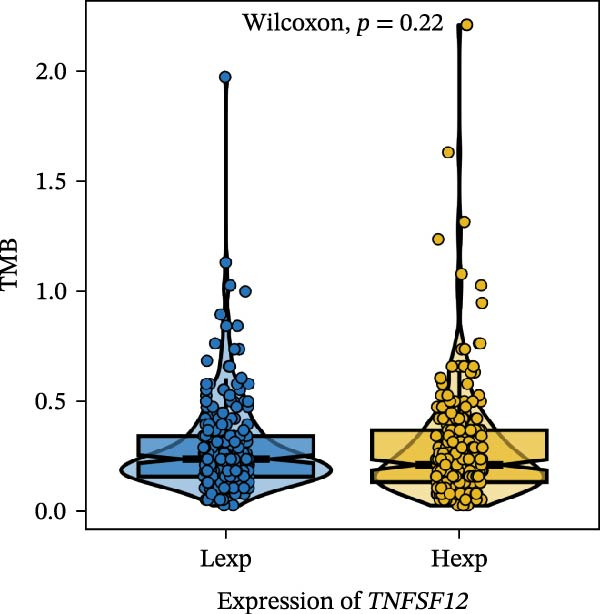
(O)

**Figure:**
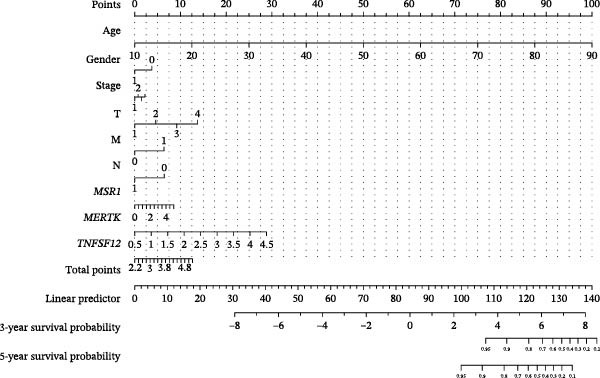
(P)

**Figure:**
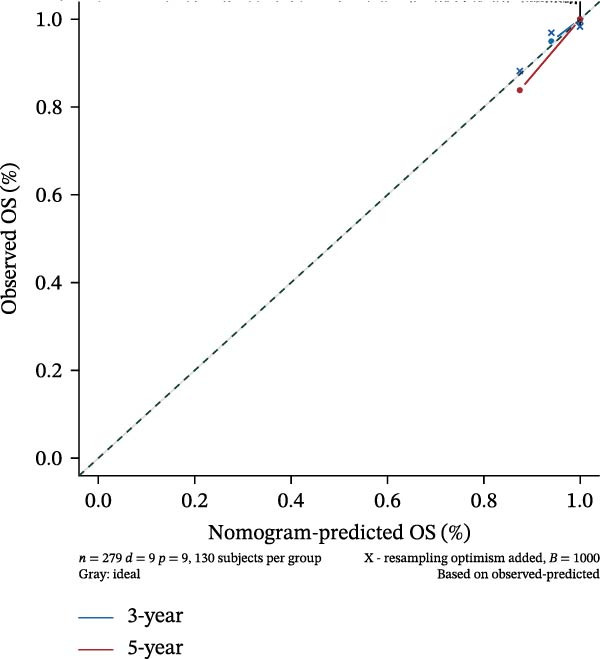
(Q)

**Figure:**
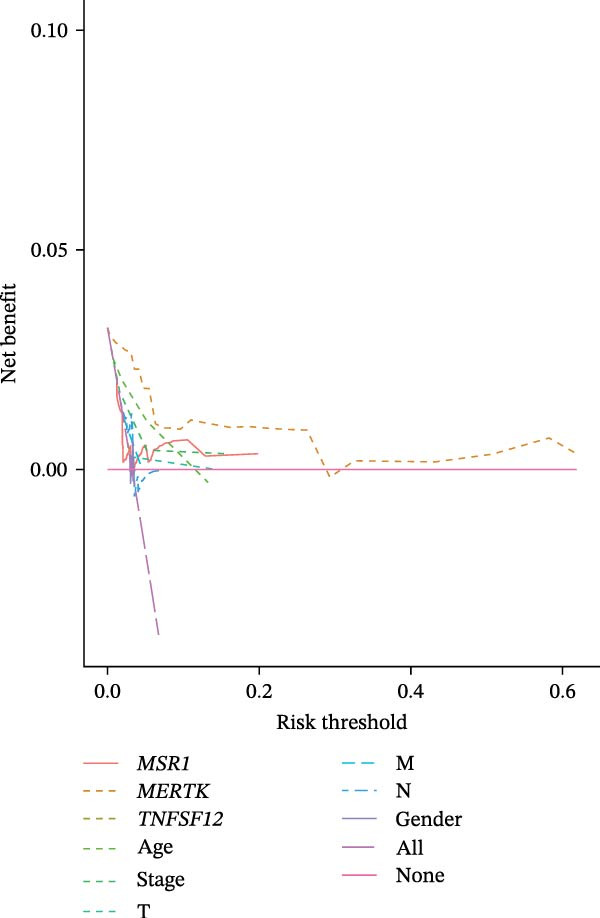
(R)

### 3.4. Multiomics Integration Reveals Gene‐Microbiome Interactions and Cellular Dynamics

The expression of *MERTK*, *MSR1*, and *TNFSF12* was associated with distinct intratumoral microbial communities (Figure 7A–C). Pearson correlation analysis identified key genus‐level associations: *MERTK* expression was positively correlated with *Haladaptatus* and Saccharibacter, but negatively with Proteus. Notably, *MSR1* showed a strong negative correlation with *Alcanivorax* and Legionella, while *TNFSF12* was negatively associated with *Alcanivorax* and Campylobacter (Figure 7D–F).

Figure 7Integrative analysis of thyroid cancer gene expression and intratumoral microbial communities. (A) *MERTK*‐microbe associations: box plots showing differential abundance of *Haladaptatus*, Proteus, and Sulfolobus in high‐expression (yellow) vs. low‐expression (blue) cohorts. (B) *MSR1*‐microbe associations: box plots revealing Lachnoclostridium and *Alcanivorax* enrichment in high‐expression (yellow) group. (C) *TNFSF12*‐microbe associations: box plots demonstrating Campylobacter and Anoxybacillus signatures in high‐expression (yellow) cohort. (D) *MERTK*‐microbe correlations: scatterplots with trend lines confirming positive (*Haladaptatus*, Tetrasphaera; blue) and negative (Anaplasma, Legionella; orange) Pearson correlations. (E) *MSR1*‐microbe correlations: scatter plots showing positive (*Haladaptatus*; blue) and negative (*Alcanivorax*, Legionella; orange) associations. (F) *TNFSF12*‐microbe correlations: scatterplots highlighting strong inverse correlation with *Alcanivorax* (orange) and positive links to Anditalea (blue).

**Figure:**
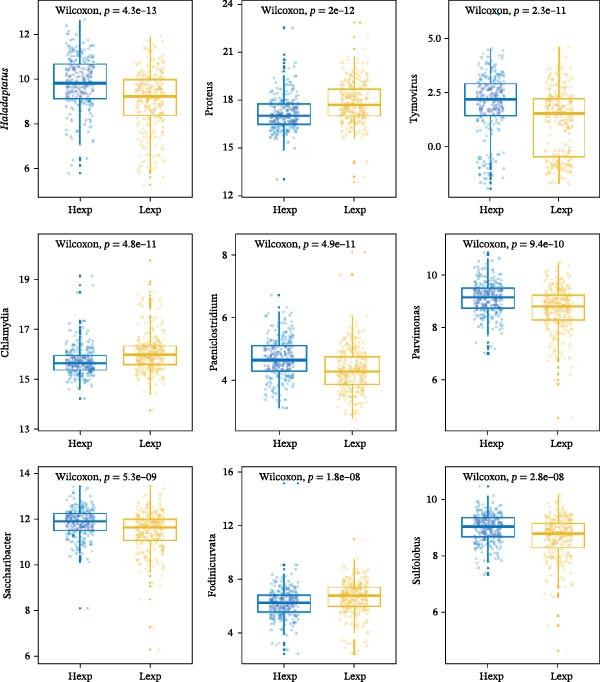
(A)

**Figure:**
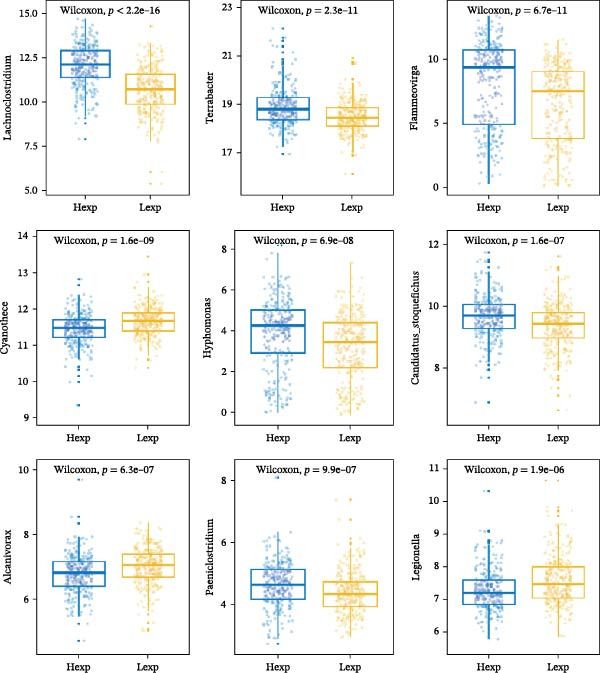
(B)

**Figure:**
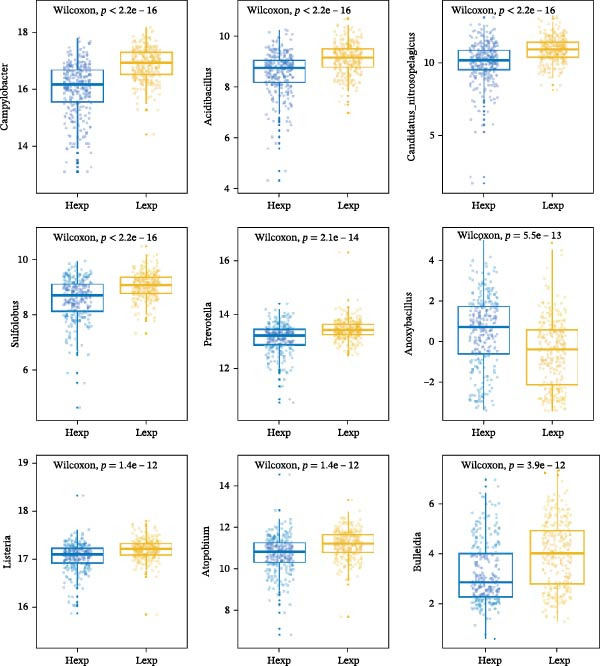
(C)

**Figure:**
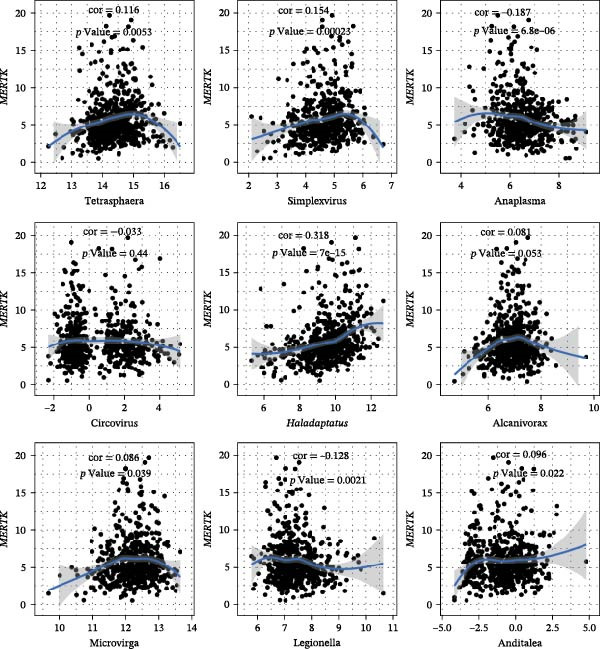
(D)

**Figure:**
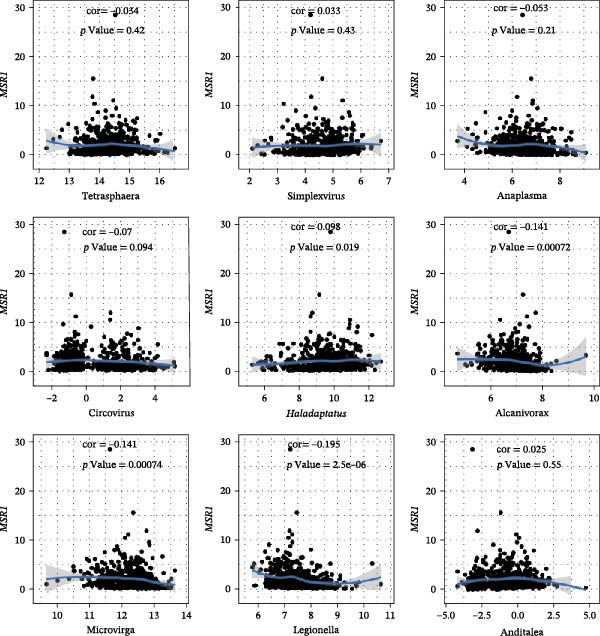
(E)

**Figure:**
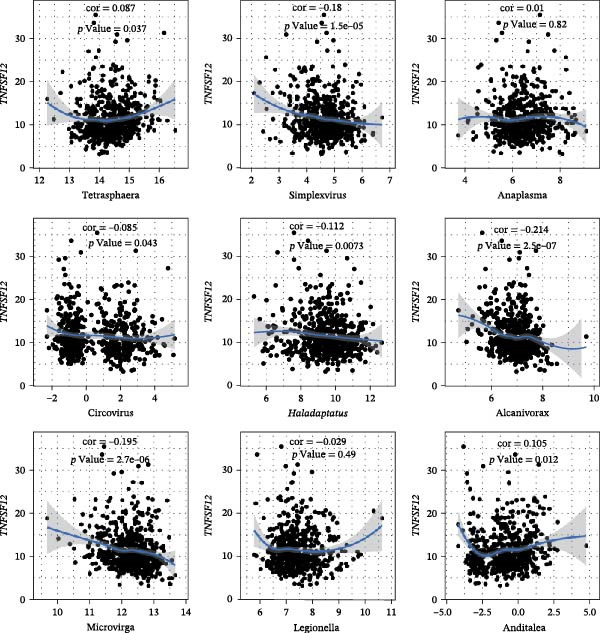
(F)

In the TME, CellCall inference highlighted significant ligand–receptor‐mediated communication involving myeloid cells (Figure 8A–B). Furthermore, pseudotemporal trajectory analysis of myeloid cells revealed dynamic, stage‐specific expression of the hub genes: *MSR1* was sustainedly upregulated in late stages, *MERTK* peaked mid‐trajectory, and *TNFSF12* exhibited fluctuating expression (Figure 8C–F).

Figure 8Single‐cell profiling reveals myeloid cell communication networks and bidirectional differentiation trajectory. (A, B) Communication networks: dot plot (A) and chord diagram (B) visualizing significant disease‐associated ligand–receptor interactions (width = interaction strength) centered on myeloid cells. (C,D) Myeloid trajectory: pseudotime trajectory by Monocle2 depicting bidirectional differentiation from a midpoint (C); cell type positioning along the trajectory (D). (E) Branch point dynamics: heatmap of genes with significant expression shifts at differentiation branch points, clustered into six functional modules. (F) Gene expression dynamics: line plots tracing *MSR1* (sustained late upregulation), *MERTK* (mid‐trajectory peak), and *TNFSF12* (fluctuating) expression along pseudotime.

**Figure:**
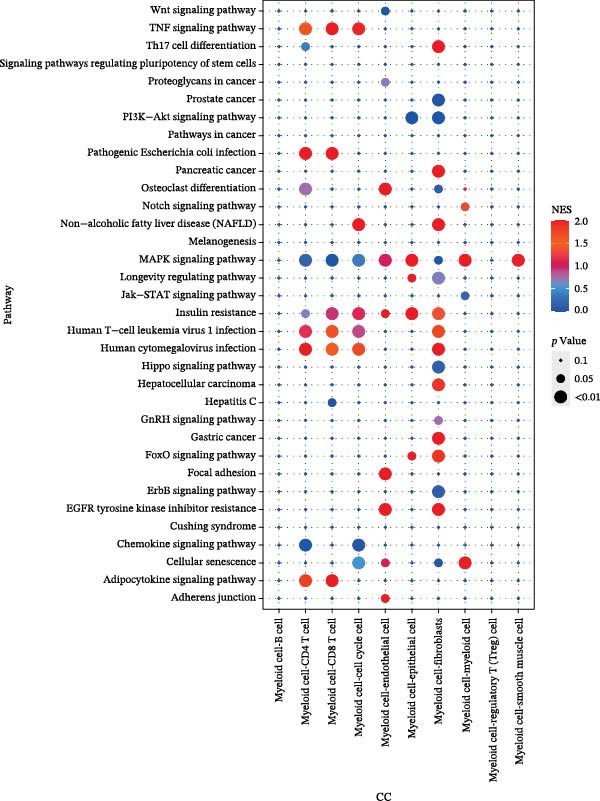
(A)

**Figure:**
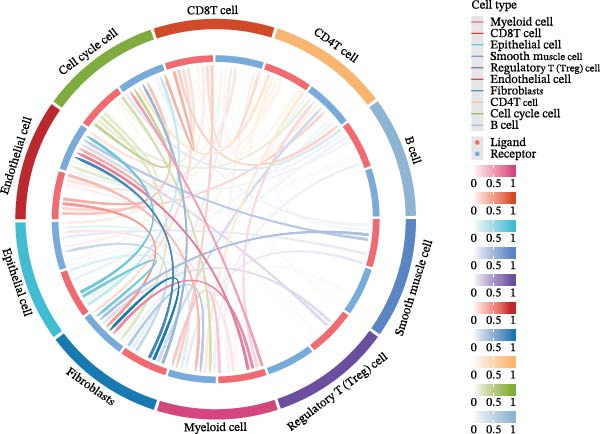
(B)

**Figure:**
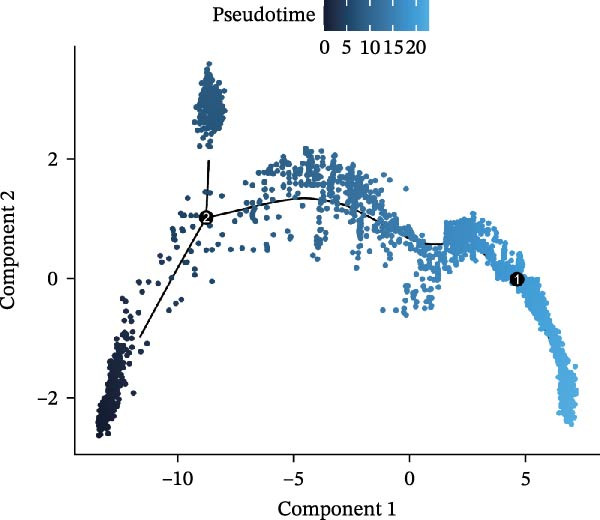
(C)

**Figure:**
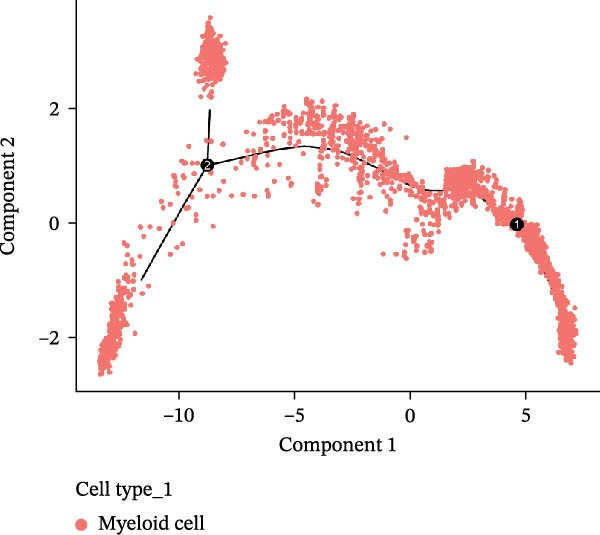
(D)

**Figure:**
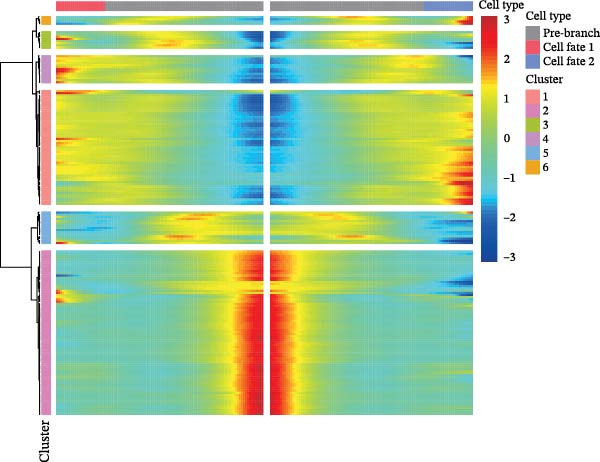
(E)

**Figure:**
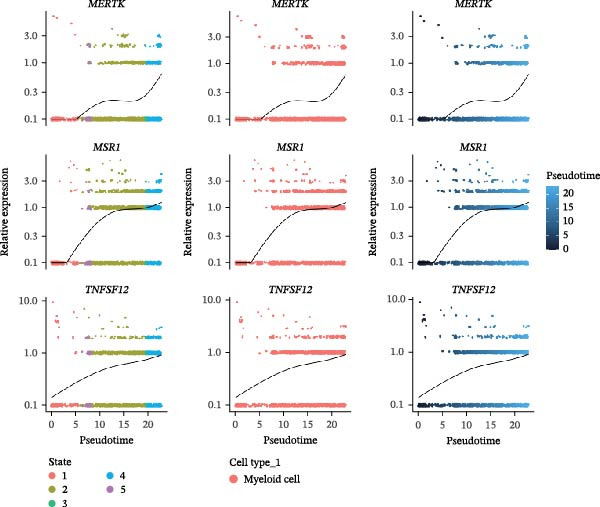
(F)

### 3.5. Experimental Validation of *TNFSF12* in Thyroid Cancer Progression

Molecular docking suggested potential interactions between *MERTK*, *MSR1*, *TNFSF12*, and selected bioactive compounds, though the observed binding energies were modest, indicating preliminary interactions that warrant further experimental validation (Figure 9A–C). Differential expression analysis of self‐collected specimens revealed no statistically significant differential expression of *MERTK*, *MSR1*, or *TNFSF12* between carcinoma (Ca) and adjacent normal (Adj) tissues (Figure 9D); however, expression levels in carcinoma samples showed a trend of increase compared to adjacent tissues across all three genes. While this trend aligns with prior high‐throughput data, it requires further validation in larger cohorts. (affinities ranging from −2.43 to −3.75 kcal mol‐^1^), while the concordant carcinoma‐upregulated expression pattern—despite lacking statistical significance—aligns with prior high‐throughput analyses, substantiating their potential as viable therapeutic targets for thyroid cancer intervention. In functional studies, *TNFSF12* was consistently and significantly upregulated across multiple thyroid cancer cell lines (TCP‐1, IHH4, and BCPAP cells) (Figure 10A, B). Gain‐and loss‐of‐function experiments demonstrated its critical role in driving malignancy: *TNFSF12* knockdown significantly suppressed cell viability, while its overexpression enhanced proliferative capacity in TPC‐1 cells (Figure 10C–F). This protumorigenic function was further validated across multiple hallmarks of cancer, as *TNFSF12* knockdown inhibited clonogenicity, invasion, and migration, whereas its overexpression potently promoted these phenotypes in TPC‐1 cells (Figure 11A–D). Collectively, these data establish *TNFSF12* as a functional oncogene in thyroid cancer.

Figure 9Molecular docking profiles and clinical expression validation of hub genes. (A) *MERTK*‐caffeine interaction: molecular model (PDB: 3 bpr) showing caffeine ligand (red) bound to *MERTK* (green ribbon) with key hydrogen bonds (yellow dashes). Binding energy: −3.75 kcal mol^−1^. (B) *MSR1*‐resveratrol interaction: structural complex (PDB: 7dpx) depicting resveratrol (red) docked to *MSR1* (green). Binding energy: −2.43 kcal mol^−1^. (C) *TNFSF12*‐sulfadimethoxine interaction: Predicted binding pose (UniProt: O43508) with sulfadimethoxine ligand (red). Binding energy: −2.94 kcal mol^−1^. (D) Clinical expression analysis: box plots comparing gene expression in carcinoma (Ca, light blue) vs. adjacent normal tissues (Adj, pink). Horizontal lines indicate higher median expression in Ca for all genes (ns = not significant).

**Figure:**
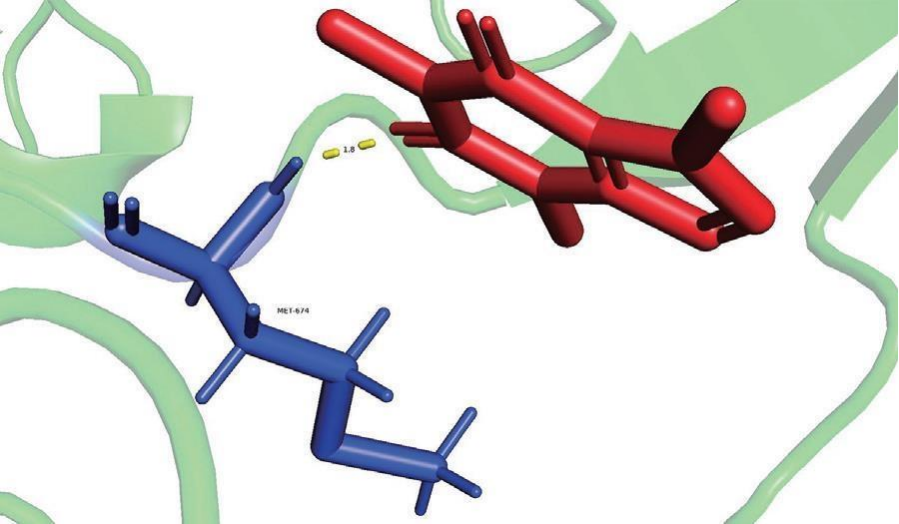
(A)

**Figure:**
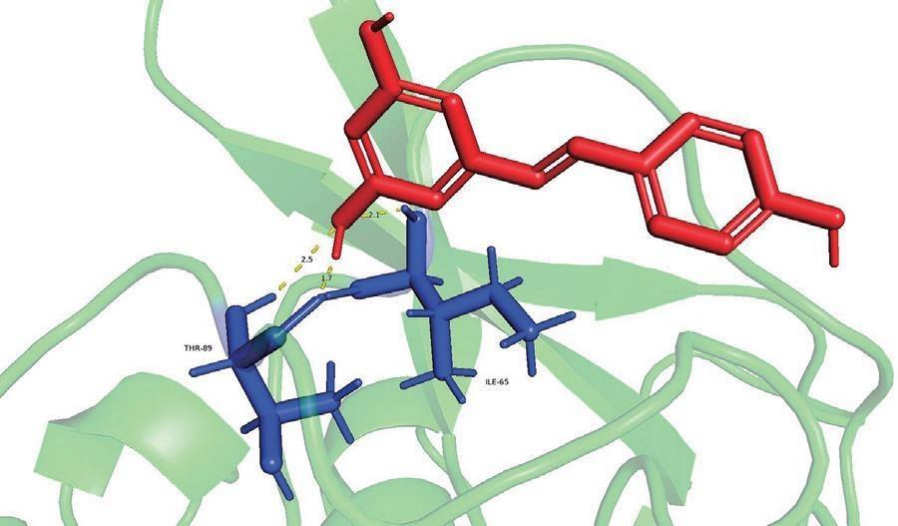
(B)

**Figure:**
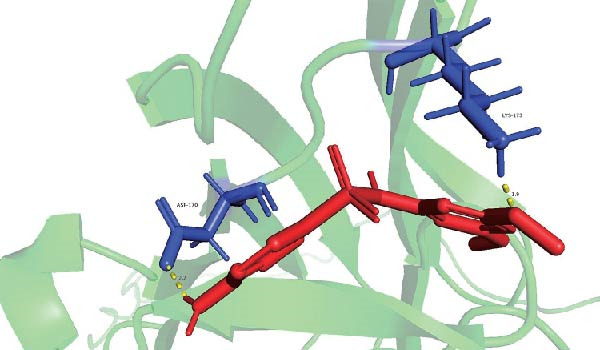
(C)

**Figure:**
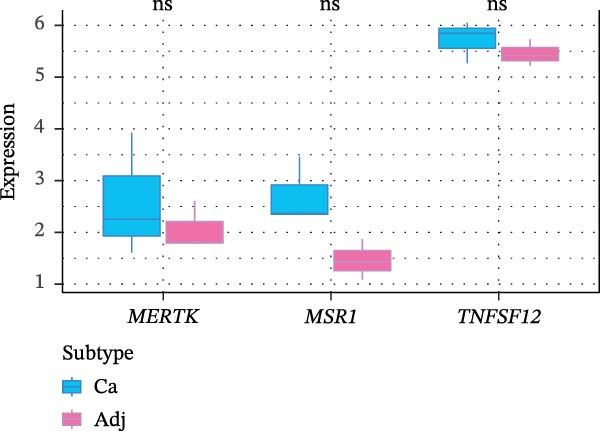
(D)

Figure 10
*TNFSF12* expression profiling in normal and malignant thyroid cell lines and its regulatory effects on cell viability via genetic knockdown and overexpression. (A, B) *TNFSF12* expression levels across cell lines (IHH4, BCPAP, and TPC‐1) and normal thyroid cell (Nthy‐ori‐3‐1) detected using seven distinct primer sets, demonstrating significant upregulation in cancer cells (*p*  < 0.05). (C) Efficient knockdown of *TNFSF12* expression via three independent siRNAs in TPC‐1 cells and IHH4 cells (*p*  < 0.001 vs. siRNA‐NC). (D) Reduced cellular viability in siRNA‐transfected thyroid cancer cells measured by CCK‐8 assay in TCP‐1 cells ( ^∗^ *p*  < 0.001 vs. siRNA‐NC). (E) Validation of *TNFSF12* overexpression efficiency using plasmid vectors in TCP‐1 cells. (F) Enhanced proliferative capacity in *TNFSF12*‐overexpressing cells by CCK‐8 assay in TCP‐1 cells ( ^∗^ *p*  < 0.001 vs. Vector‐NC). Data presentation and statistics data are expressed as mean ± SD from three independent experiments. Statistical significance: *p* < 0.0001, *p* < 0.001, *p* < 0.05 versus the Nthy‐ori‐3‐1 group, siRNA‐NC group, or vector‐NC group.  ^∗∗∗^ *p* < 0.001, 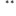 *p* < 0.01, and 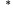 *p* < 0.05.

**Figure:**
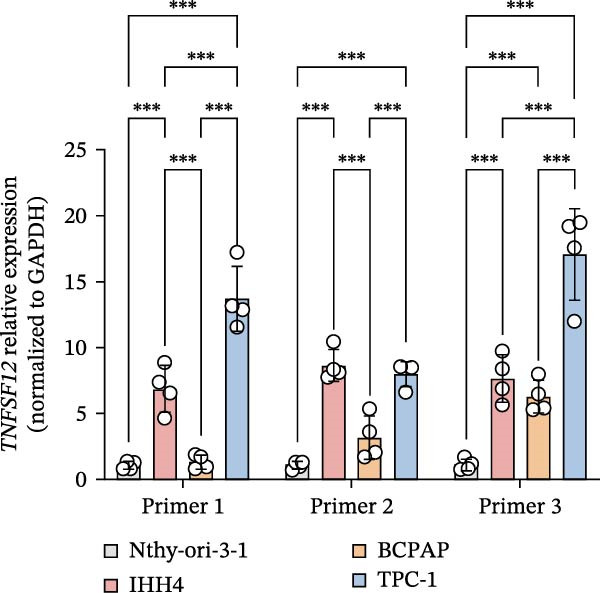
(A)

**Figure:**
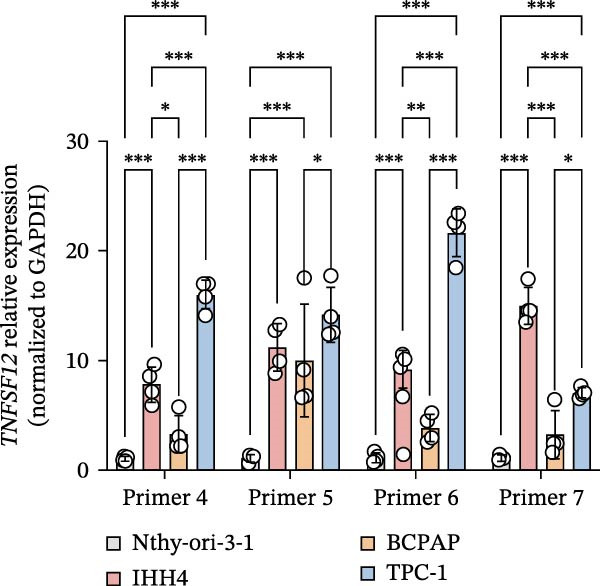
(B)

**Figure:**
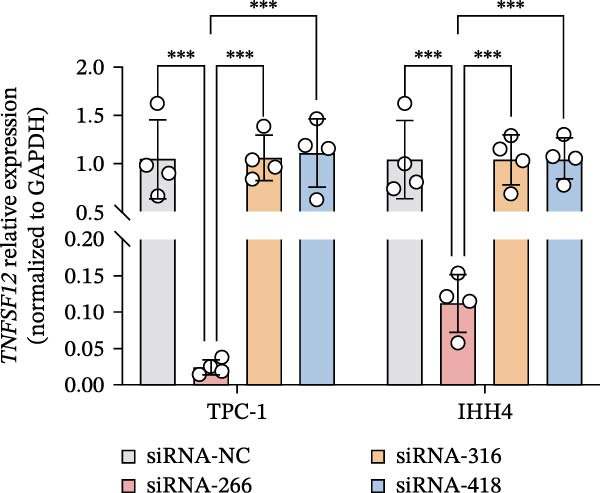
(C)

**Figure:**
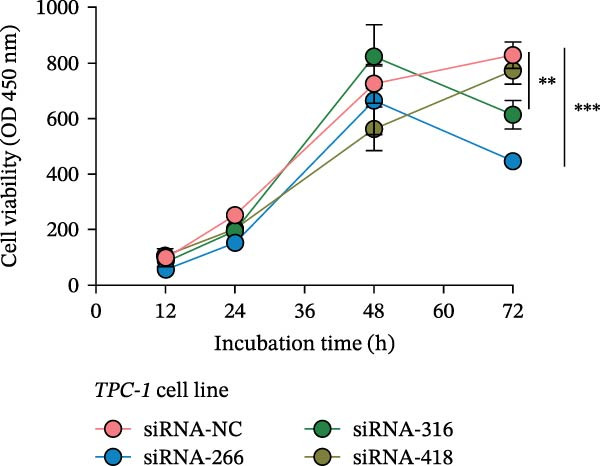
(D)

**Figure:**
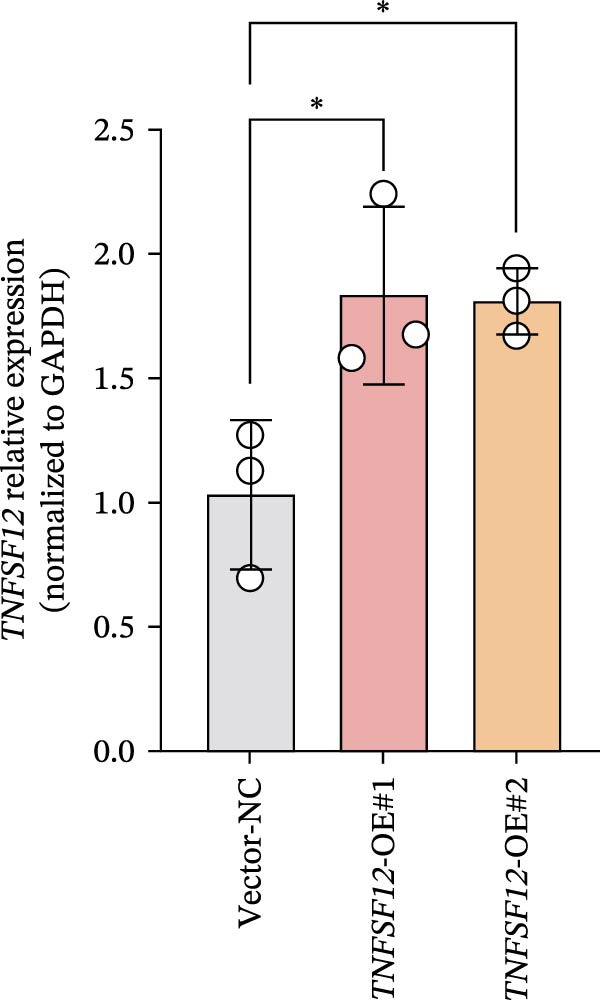
(E)

**Figure:**
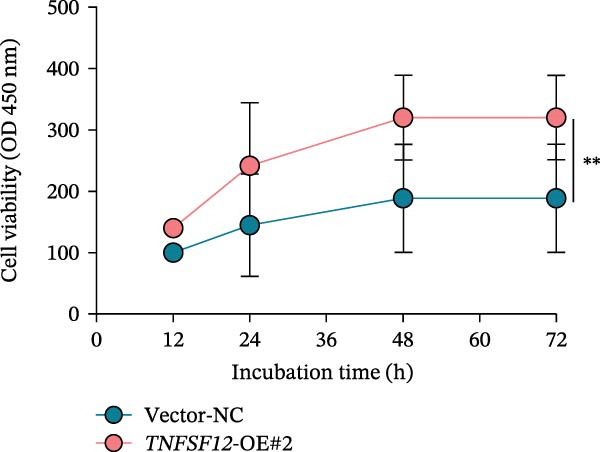
(F)

Figure 11Effects of *TNFSF12* on colony formation, invasion, migration and statistical analysis in in TCP‐1 cells. (A) Colony formation assay; (B) transwell assay; (C) wound healing assay; (D) statistical analysis of functional metrics. Data are presented as mean ± SD of three independent experiments. Statistical significance is denoted as *p* < 0.0001, *p* < 0.001, and *p* < 0.05 versus the siRNA‐NC or vector‐NC control groups.  ^∗∗∗^ *p* < 0.001,  ^∗∗^ *p* < 0.01, and  ^∗^ *p* < 0.05.

**Figure:**
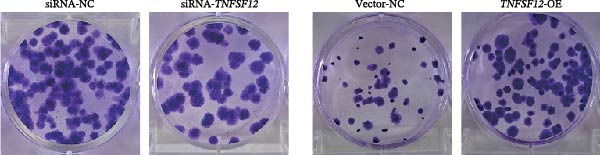
(A)

**Figure:**
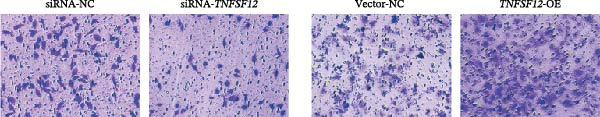
(B)

**Figure:**
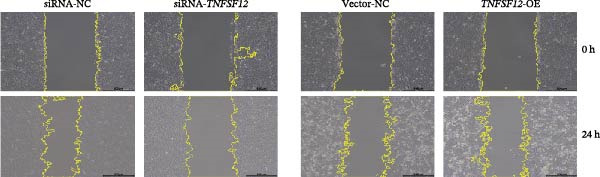
(C)

**Figure:**
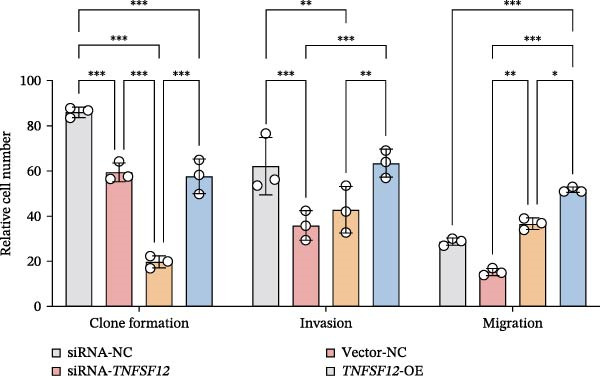
(D)

## 4. Discussion

The TME of thyroid cancer is highly heterogeneous, with myeloid cells playing pivotal yet incompletely understood roles in immune modulation and disease progression [11]. By integrating single‐cell transcriptomics, multiomics data, and MR, we constructed a multitiered regulatory framework that connects a tumor‐enriched myeloid subpopulation to causally implicated hub genes (*MERTK*, *MSR1*, and *TNFSF12*) and ultimately to diverse clinical and molecular phenotypes. This integrative approach not only delineates the cellular and genetic architecture of the myeloid‐driven TME but also reveals the complex, context‐dependent functionality of key regulators, particularly *TNFSF12*.

At the functional level, each of these three genes exerts distinct mechanisms that influence tumor progression. *MERTK* promotes cell cycle activation through the Wnt/β‐catenin signaling pathway and shows a positive correlation with TMB and specific commensal microbes such as *Haladaptatus* [12]. *MSR1* activates proinflammatory cascades via the *TLR/NF-κB* pathway while simultaneously suppressing potentially protective microbiota taxa, including *Alcanivorax*, thereby maintaining an immunosuppressive and dysbiotic TME [13]. In contrast, *TNFSF12* demonstrates the ability to inhibit BRAF mutations and is associated with improved response to immunotherapy, suggesting a context‐dependent tumor‐suppressive function [14–18]. Notably, molecular docking analysis confirms the binding potential between *MSR1* and resveratrol, highlighting a promising therapeutic avenue. These mechanistic insights reveal the multidimensional regulatory networks mediated by these genes, encompassing immune regulation, microbial ecology, and cellular signaling pathways.

A particularly intriguing finding pertains to the inconsistent expression pattern and context‐dependent function of *TNFSF12*. Contrary to certain bioinformatic predictions suggesting its downregulation in thyroid cancer, experimental validation across multiple cell lines (IHH4, BCPAP, TPC‐1) consistently revealed overexpression of *TNFSF12*, with functional assays confirming its role in promoting cell proliferation, invasion, and migration. This discrepancy highlights the complex, and possibly cell type‐specific, functionality of *TNFSF12*—its effects may be protective effects at the genetic level in immune cells while directly driving tumorigenic behaviors in epithelial contexts [19–23]. The seemingly contradictory roles of *TNFSF12*—identified as a protective factor in MR analysis yet exhibiting protumorigenic functions in vitro—may reflect its cell type‐specific and context‐dependent nature. This duality aligns with the pleiotropic functions of the *TWEAK/Fn14* (*TNFSF12*/*TNFRSF12A*) axis, which is known to play dual roles in tissue repair and cancer progression depending on cellular context [24]. Similarly, the observed correlations between *MSR1* and depletion of *Alcanivorax*, as well as between *MERTK* and enrichment of *Haladaptatus*, suggest a novel “gene‐microbe” coregulation axis warranting further investigation. These findings underscore the intricate and sometimes paradoxical nature of gene function within the TME.

The theoretical implications of this study extend current models of thyroid cancer by integrating multiomic interactions among immune genes, microbial communities, and tumor cells. *MSR1* exemplifies dual modulation of both immune and microbial components, whereas *MERTK* illustrates the synergy between oncogenic signaling pathways and microbiome composition. The context‐dependent functionality of *TNFSF12* challenges the conventional “single‐gene, single‐function” paradigm and underscores the necessity of incorporating cellular origin and microenvironmental context into mechanistic frameworks. Although a preliminary clinical predictive model has been developed using these multiomics features, several limitations remain, including a small single‐cell sample size, reliance on public microbiome databases, and the absence of cell‐type‐specific functional validation for *TNFSF12*. These limitations highlight specific directions for future research to validate and expand upon the proposed model.

In summary, this integrated multiomics study elucidates the central role of myeloid cell subpopulations and their key regulatory genes—*MERTK*, *MSR1*, and *TNFSF12*—in shaping the immune and microbial landscape of thyroid cancer. By integrating single‐cell profiling, genetic causal inference, and functional assays, we propose a comprehensive target axis linking myeloid subsets, gene modules, and clinical outcomes. The paradoxical behavior of *TNFSF12* across genetic and functional levels highlights the complexity of the thyroid cancer microenvironment, indicating that its function may be highly context‐dependent. These findings not only enhance our understanding of thyroid cancer heterogeneity but also establish a theoretical foundation for the development of targeted immunotherapies and personalized treatment strategies.

## Author Contributions

Hong Qiao and Junjie Yu conceived and designed the study. Junjie Yu, Jingjing Li, and Lilan Wang carried out the experiments. Hong Qiao analyzed the experimental data. Hong Qiao and Junjie Yu wrote and revised the manuscript and confirm the authenticity of all the raw data. All authors are involved in the production of the article.

## Funding

The authors have nothing to report.

## Disclosure

All authors have read, agreed, and approved the final manuscript.

## Ethics Statement

This study was reviewed and approved by the Ethics Committee of the Second Affiliated Hospital of Harbin Medical University (Approval Number: YJSKY2025‐282). The use of human tissue samples complied with all relevant ethical regulations, and informed consent was obtained from all participants.

## Conflicts of Interest

The authors declare no conflicts of interest.

## Supporting Information

Additional supporting information can be found online in the Supporting Information section.

## Supporting information


**Supporting Information 1** Table S1. siRNA sequence information. Lists the sense and antisense strand sequences for three independent siRNA duplexes used to knockdown TNFSF12, along with the sequence for the negative control.

**Supporting Information 2** Table S2. qPCR primer sequences. Lists multiple forward and reverse primer pairs used for detecting TNFSF12 gene expression, as well as the primer sequences for the reference gene GAPDH.

**Supporting Information 3** Table S3. List of 100 genes from the “yellow” coexpression module. Contains the complete set of genes identified within the disease‐associated yellow module via hdWGCNA analysis, which includes the three hub genes of interest (*MERTK*, *MSR1*, and *TNFSF12*).

**Supporting Information 4** Table S4. Results of heterogeneity tests for the Mendelian Randomization analysis. Shows the *Q* statistic, degrees of freedom, and *p*‐value for MR analyses of the three genes (*MERTK*, *MSR1*, *TNFSF12*) using both the MR‐Egger and inverse‐variance weighted methods, indicating no significant heterogeneity (all *p* > 0.05).

## Data Availability

The data that support the findings of this study are available upon request from the corresponding author. The data are not publicly available due to privacy or ethical restrictions.
