# Supplementary material for: Investigating the Role of TNFSF12 in Thyroid Cancer Progression via Single‐Cell RNA Sequencing and Integrated Multiomics Analyses
Source: Mediators Inflamm. 2026 Apr 3;2026:4753653. doi: 10.1155/mi/4753653 (PMC13051803; doi:10.1155/mi/4753653)
Supplement: Supplementary file 1 — Supporting Information 1 Table S1. siRNA sequence information. Lists the sense and antisense strand sequences for three independent siRNA duplexes used to knockdown TNFSF12, along with the sequence for the negative control. [file MI-2026-4753653-s001.docx]

Supplement Table 2. siRNA sequence

| DUplexName | SenseSeq5'→3' | AntiSeq5'→3' |
| --- | --- | --- |
| hTNFSF12-266 | AGGAGGAGCUGGUGGCAGA | UCUGCCACCAGCUCCUCCU |
| hTNFSF12-418 | GCAGCCCAUUAUGAAGUUCAU | UGAACLUCAUAAUGGGCUGC |
| hTNFSF12-316 | CAGACAGAAGAAAGCCAGGAU | AUCCUGGCUUUCUUCUGUCUG |
| Negative control | UCUCCGAACGUGUCACGUTT | ACGUGACACGUUCGGAGAATT |
