# Supplementary material for: Investigating the Role of TNFSF12 in Thyroid Cancer Progression via Single‐Cell RNA Sequencing and Integrated Multiomics Analyses
Source: Mediators Inflamm. 2026 Apr 3;2026:4753653. doi: 10.1155/mi/4753653 (PMC13051803; doi:10.1155/mi/4753653)
Supplement: Supplementary file 2 — Supporting Information 2 Table S2. qPCR primer sequences. Lists multiple forward and reverse primer pairs used for detecting TNFSF12 gene expression, as well as the primer sequences for the reference gene GAPDH. [file MI-2026-4753653-s004.docx]

Supplement Table 1. primer sequence

| Gene_symbol | Forward Primer​ | Reverse Primer |
| --- | --- | --- |
| TNFSF12 | CGCCTTTCCTGAACCGACTA | CACTCACTGTCCCGTCCACA |
|  | AGGTGTCTGGGCTGTTGGC | TGGAAGAGTCCGAAGTAGGTGA |
|  | AGGTGTCTGGGCTGTTGGC | GAAGAGTCCGAAGTAGGTGAGG |
|  | CGCCTTTCCTGAACCGACTA | TTGATTCTGGCTTCCTCCC |
|  | CGCCTTTCCTGAACCGACTA | GCTGTTGATTCTGGCTTCCTC |
|  | ATCGCAGCCCATTATGAAGT | CCCGATCTGGCGGTTGTAG |
| GAPDH | ACAACAGCCTCAAGATCATCAGC | GCCATCACCCCACAGTTTCC |
