# Supplementary material for: Investigating the Role of TNFSF12 in Thyroid Cancer Progression via Single‐Cell RNA Sequencing and Integrated Multiomics Analyses
Source: Mediators Inflamm. 2026 Apr 3;2026:4753653. doi: 10.1155/mi/4753653 (PMC13051803; doi:10.1155/mi/4753653)
Supplement: Supplementary file 3 — Supporting Information 3 Table S3. List of 100 genes from the “yellow” coexpression module. Contains the complete set of genes identified within the disease‐associated yellow module via hdWGCNA analysis, which includes the three hub genes of interest (MERTK, MSR1, and TNFSF12). [file MI-2026-4753653-s003.docx]

Supplement Table 3. 100 genes from the yellow module

|  | | | | |
| --- | --- | --- | --- | --- |
| C1QC | ITM2B | FOLR2 | LINC02712 | FCGBP |
| C1QA | CSF1R | IGSF21 | CPM | ITPR2 |
| C1QB | FCGR2A | FCHO2 | CEBPD | CD302 |
| GPR34 | TPT1 | A2M | SELENOP | IL2RA |
| MS4A7 | MSR1 | CTSD | GIMAP4 | HNMT |
| SLC1A3 | VSIG4 | KCTD12 | CD37 | CSGALNACT1 |
| DAB2 | OLFML3 | STMN1 | TNFSF12 | ADORA3 |
| SLC40A1 | MARCKS | WASF2 | STAB1 | PDGFC |
| FCGR3A | TREM2 | DDIT4 | MFSD1 | DRAM2 |
| MS4A4A | SESN1 | FCGR1A | LAPTM4A | ISCU |
| LTC4S | AKR1B1 | RB1 | MAF | EPB41L2 |
| GGTA1 | RASSF4 | ADAP2 | CD84 | GIMAP1 |
| TMEM176B | TBXAS1 | PIK3R1 | HCLS1 | HOMER3 |
| CD163 | CD14 | LILRB4 | PLTP | APBB1IP |
| PDK4 | MAFB | CREG1 | F13A1 | GPR82 |
| C3AR1 | SRGAP1 | RNF130 | SIGLEC10 | HLA-E |
| LAIR1 | LPAR6 | MERTK | SCN1B | EBI3 |
| SLCO2B1 | APOE | CNPY3 | PDGFB | CYB5R1 |
| CTSC | MRC1 | KCNMA1 | LHFPL2 | LINC01094 |
| ARHGAP18 | CTSB | TMEM176A | CHCHD10 | SERPINB6 |
