# Supplementary material for: Investigating the Role of TNFSF12 in Thyroid Cancer Progression via Single‐Cell RNA Sequencing and Integrated Multiomics Analyses
Source: Mediators Inflamm. 2026 Apr 3;2026:4753653. doi: 10.1155/mi/4753653 (PMC13051803; doi:10.1155/mi/4753653)
Supplement: Supplementary file 4 — Supporting Information 4 Table S4. Results of heterogeneity tests for the Mendelian Randomization analysis. Shows the Q statistic, degrees of freedom, and p‐value for MR analyses of the three genes (MERTK, MSR1, TNFSF12) using both the MR‐Egger and inverse‐variance weighted methods, indicating no significant heterogeneity (all p > 0.05). [file MI-2026-4753653-s002.docx]

Supplement Table 4. Heterogeneity test of the three genes

| **Gene** | **id.exposure** | **Method** | **Q value** | **Q_df** | **P value** | **Heterogeneity（α=0.05）** |
| --- | --- | --- | --- | --- | --- | --- |
| MERTK | eqtl-a-ENSG00000153208 | MR Egger | 5.127 | 7 | 0.644 | P > 0.05 |
|  |  | Inverse variance weighted | 5.147 | 8 | 0.742 | P > 0.05 |
| MSR1 | eqtl-a-ENSG00000038945 | MR Egger | 0.240 | 4 | 0.993 | P > 0.05 |
|  |  | Inverse variance weighted | 1.358 | 5 | 0.929 | P > 0.05 |
| TNFSF12 | eqtl-a-ENSG00000239697 | MR Egger | 0.903 | 2 | 0.637 | P > 0.05 |
|  |  | Inverse variance weighted | 1.597 | 3 | 0.660 | P > 0.05 |
